# Conventions and research challenges in considering trust with socially assistive robots for older adults

**DOI:** 10.3389/frobt.2025.1631206

**Published:** 2025-11-26

**Authors:** Aisha Gul, Liam Turner, Carolina Fuentes

**Affiliations:** School of Computer Science and Informatics, Cardiff University, Cardiff, United Kingdom

**Keywords:** systematic literature review, socially assistive robots, trust, older adults, elderly, robots

## Abstract

**Introduction:**

The global ageing population rise creates a growing need for assistance and Socially Assistive robots (SARs) have the potential to support independence for older adults. However, to allow older adults to benefit from robots that will assist in daily life, it is important to better understand the role of trust in SARs.

**Method:**

We present a Systematic Literature Review (SLR) aiming to identify the models, methods, and research settings used for measuring trust in SARs with older adults as population and analyse current factors in trust assessment.

**Result:**

Our results reveal that previous studies were mostly conducted in lab settings and used subjective self-report measures like questionnaires, interviews, and surveys to measure the trust of older adults in SARs. Moreover, many of these studies focus on healthy older adults without age-related disabilities. We also examine different human-robot trust models that influence trust, and we discuss the lack of standardisation in the measurement of trust among older people in SARs.

**Discussion:**

To address the standardisation gap, we developed a conceptual framework, Subjective Objective Trust Assessment HRI (SOTA-HRI), that incorporates subjective and objective measures to comprehensively evaluate trust in human-robot inter-actions. By combining these dimensions, our proposed framework provides a foundation for future research to design tailored interventions, enhance interaction quality, and ensure reliable trust assessment methods in this domain. Finally, we highlight key areas for future research, such as considering demographic sensitivity in trust-building strategies and further exploring contextual factors such as predictability and dependability that have not been thoroughly explored.

## 1 Introduction

Across the globe, people are living longer, with most people anticipated to live into their 60s and beyond. As a result, the total population and percentage of older adults in the world are increasing - with one in six expected to be aged 60 or over by 2030 and nearly half associate themselves with some kind of age-related disability (World Health Organisation, 2022). As demographics change, there is a need to better support the ageing population; society and services must be prepared to support longer independent living, ensure quality of life, and have healthcare systems that are able to provide interventions aimed at mitigating or managing health problems affecting older people (e.g., frailty, disabilities, loneliness). This demographic change presents three main challenges. Firstly, providing healthcare assistance can be costly (IMF- Cost of Ageing, 2023). For example, in the UK, the total expenditure of taking care of older adults (including hospital and community health services, family health services, and pharmaceutical services) runs in thousands of pounds per year (UKHSA-UK Gov, 2023). According to the UK Office for Budget Responsibility (OBR) (UKHSA-UK Gov, 2023), health spending per person generally increases with age.

Secondly, older adults may resist the idea of someone assisting them (e.g., for privacy reasons). According to Alzheimer’s Association (Alzheimer’s Society, 2023), 70% of adults worry about being a burden on their children. Finally, it may also hurt the self-esteem of some older adults to ask for help. For all these reasons, it is important to devise interventions to enable older adults to live independently and to be active members of society for a longer time (Motamed-Jahromi and Kaveh, 2021).

Assistive technologies (ATs) can support people with disabilities and/or impairments to complete Activities of Daily Living (ADL) which might otherwise be difficult or impossible (UK Government assistive technology, 2023). Thus, ATs have multiple benefits: they promote people’s active engagement in ADLs (such as employment and education), encourage people’s independence, lessen the need for carers, and lower social and healthcare expenses (WHO Assistive Technology, 2023). ATs allow older adults to maintain or enhance their functioning and independence, enabling them to perform ADLs with ease, contributing to maintaining or improving their independence. Traditional ATs commonly used by older adults include: *self-care:* as shoe removal aids, long handle shoe-horns, bathtub bench; *mobility:* as walking canes, scooters, prostheses; *communication:* as hearing aids, talking devices, tablets/computers, etc., and *safety:* as grab bars, pill organizer, wheelchair ramps, etc., (AssistiveTechnology, 2018).

In order to take full advantage of any new technology and use it to its full extent, it is important that people trust it. According to Bok (1999), “Whatever matters to human beings, trust is the atmosphere in which it thrives.” For example, we trust a new car to function properly so we can safely travel in it (Holzner, 1972). Recently, Socially Assistive Robots (SARs) (Eftring and Frennert, 2016) have emerged as a new technology for assisting old people in their daily lives. These robots are capable of assisting in a variety of activities including mobility, housekeeping, medication management, eating, grooming, bathing, and social communications (Cowan et al., 2012). To fully understand how older adults feel in the presence of a SAR, trust is an important element to consider because the level of trust may impact the ultimate engagement and effectiveness of a SAR (for Computing Machinery, 2012). Decline in cognition (e.g., memory, problem-solving, decision making) is expected with ageing, especially for tasks that require one to quickly process information (Murman, 2015; Ye and Kankanhalli, 2017). Therefore, the way in which trust is established between older people and SARs are worthy of deeper discussion and it can contribute to better meeting the needs of older people.

As the development of SARs move towards providing seamless human-like interactions, the extent of trust older adults place in these technologies may directly affect not only the support they receive but also the overall acceptability of SARs in their daily lives (McMurray et al., 2017; Zafrani et al., 2023). The level of trust placed in SARs could potentially play a pivotal role in shaping the overall assistance and engagement experienced by older individuals (Zafrani et al., 2023). For example, if individuals have a high level of trust in SARs and feel comfortable interacting with them, they are more likely to use SARs in healthcare or other applications, that potentially can improve their wellbeing.

Therefore, it is important to understand trust between human and SARs for making best use of SARs for assisted living (Schwaninger et al., 2021). Existing studies in the field of Human-Robot Interaction (HRI) have extensively investigated acceptance and perception of older adults with robots more generally (Naneva et al., 2020; Vandemeulebroucke et al., 2021; Savela et al., 2018). However, there has been limited explicit focus in the literature surrounding understanding the role of trust in the context of SARs specifically for older adults. For example, Campagna and Rehm (2025) conducted a broad review of trust in HRI across industrial and social-care domains, highlighting key trust factors and emerging methods such as sensor-based assessment, but without further exploring the contextual considerations of older adult populations.

We conducted a systematic literature review (SLR) that focuses on the relationship between trust and older adults in the context of SARs. The objective of this SLR is to compile and analyze the existing research that has explored trust of older adults in SARs. The research questions guiding this review are:

RQ1. What are the methodologies and metrics used to assess trust in the interaction with SARs?
RQ2. What types and categories of SARs are studied in trust research studies, and how do their features shape experimental design?
RQ3. What are the research environments and factors influencing trust in SARs and which factors have been under explored?
RQ4. Which demographics have studies measured trust in SARs, which are underrepresented, and what population sizes are studies using?

By systematically reviewing a wide range of studies conducted between 2013 and 2024, we are able to identify and synthesize the methods employed to measure trust in SARs for older adults. Through our review, we found that questionnaires, discussions, interviews, and surveys were commonly used methods to evaluate the level of trust in SARs. However, it is important to note that many of these studies focused on a population without age-related disabilities, raising the question of whether these methods are equally applicable to older adults with such disabilities.

Our findings highlight the need for standardized approaches in measuring trust in SARs, considering the unique challenges and needs of older adults. Additionally, we emphasize the importance of evaluating trust in real-world environments such as older adults’ homes, also considering different demographic backgrounds, to capture a more accurate reflection of their trust levels. Moreover, we advocate for increased involvement of older adults with age-related disabilities in future research to better understand their trust dynamics and tailor SARs to their specific needs. This literature review sets the stage for further research and offers valuable insight into the measurement, factors and implications of trust in the context of SARs, ultimately facilitating the development of trustworthy and effective robotic solutions for older adults.

Next sections are organized as follows. In Section 2, we discuss the key concepts related to trust in SARs. In Section 3, we describe the methodology and the search strategy we adopted to conduct our SLR. In Section 4, we present our results, while, in Section 5, we present a detailed discussion of the results and potential future directions. Finally, we provide our conclusions in Section 6.

## 2 Background

In this section, we introduce and discuss the key concepts of our literature review, i.e., *trust* and *SARs*, and why trust is an important consideration for SARs.

### 2.1 Socially assistive robots

Socially Assistive Robots (SARs) are a type of robot which assist humans through social interaction (Bedaf et al., 2015). They can serve as companions, pets, or service robots (Matarić and Scassellati, 2016). According to Broekens et al. (2009), SARs are understood as social entities that can communicate with users. Based on the type of assistance they provide, SARs can be categorized as either *contact assistive robots* or *social interactive robots*. While *contact assistive robots* provide physical assistance, *social interactive robots* provide assistance through social interaction Feil-Seifer and Mataric (2005). A similar categorization is suggested by Fracasso et al. (2022) and Heerink et al. (2010), who categorize SARs as either *service robots* or *companion robots*. *Service robots* help in assisting with a variety of physical activities, such as carrying heavy loads or walking assistance. RIBA robot (2023), with its human-type arms is an example of a service robot which is designed to help patients with lifting and moving heavy objects. RIBA is also capable of moving patients between a bed and a wheelchair (Joseph et al., 2018). On the other hand, *Companion robots* provide social interaction for emotional, social, or psychological support (Fracasso et al., 2022).

Studies have shown that companion robots are particularly useful for older people as they can reduce stress (Saito et al., 2003), depression (Wada et al., 2005), regulate blood pressure (Robinson et al., 2015), and improve people’s mood (Wada et al., 2003). Companion robots are available in different forms and shapes such as pet-like (e.g., pet robots) and human-like (e.g., humanoids). PARO (2023), a robotic baby seal pet, is a popular pet robot which carries various sensors to sense touch, sounds, and visual objects (Vitanza et al., 2019). Similarly, Pepper robot (2024) is a popular humanoid companion robot. Pearl (Pineau et al., 2003) is another popular companion robot which assists older patients by helping with ADL such as giving reminders about medication and appointments and using motion sensors to detect falls and physical inactivity (BUDDY, 2023).

### 2.2 Trust in different types of interactions

In this section, we first discuss the importance of trust and the factors that influence trust in human-human interactions (HHI) and human-robot interactions (HRI). We then consider trust of older adults on robots and discuss the different factors that are uniquely important in such interactions.

#### 2.2.1 Human-human interaction

In HHI, trust is fundamental to building and maintaining positive relationships. It is integral to all human interactions (PsychologyToday, 2023) and has been identified as an important foundation for interpersonal cooperation (McAllister, 1995). It fosters cooperation, communication, and emotional connection Mayer et al. (1995). Moreover, trust forms the basis of social cohesion and facilitates the smooth functioning of societies (Coleman, 1994). For example, in a work environment, higher levels of trust between an employer and an employee are linked with higher levels of performance (Alfes et al., 2012). In healthcare, lack of trust in doctors may discourage patients from benefiting from their professional advice (PsychologyToday, 2023). For example, Dang et al. (2017) found that patients with greater trust in healthcare providers were significantly more likely to complete a follow-up visit, take their medicines, and remain in care. In HHI, typically, trust depends on factors such as ability, reliability, honesty, and integrity (Malle and Ullman, 2021).

#### 2.2.2 Human-robot interaction

Nowadays, robots and other autonomous systems offer potential benefits by assisting humans in accomplishing their tasks (Lewis et al., 2018). However, to fully utilize the potential of robots, establishing trust in them is important (Campanozzi et al., 2019). Many researchers have highlighted that a comprehensive conceptualization of trust is important when designing robots that interact socially with humans as trust is integral for the acceptance and inclusion of a robot in human’s daily lives (Papadopoulos et al., 2018; Kok and Soh, 2020; Lazányi and Hajdu, 2017). Hence, humans are unlikely to use robots if they perceive the robot as untrustworthy. While trust can induce cooperation between humans and robots, building trust is extremely difficult as misaligned trust towards a robot can lead to the misuse or disuse of a robot (Rhim et al., 2023). In the context of HRI, trust extends beyond factors like the robot’s ability and reliability. It is also linked to factors such as acceptance, cooperation, effective task performance, and the overall positive experiences of users. The dynamics of trust in HRI encompass a broader spectrum of factors that go beyond the traditional criteria observed in HHI. Moreover, trust is methodologically challenging to tackle and certainly difficult to quantify and define (Salem et al., 2015), and it may come with pitfalls. In a study by Salem et al. (2015), participants followed a robot’s instructions not only because of actual trust, but also because of their enthusiasm about participating in a scientific experiment, further considering the robot to be an extension of researchers (Salem et al., 2015). According to Rhim et al. (2023) and Malle and Ullman (2021), trust in HRI is a multifaceted concept with many layers and a dynamic process that fluctuates over time.

##### 2.2.2.1 The dynamic nature of trust

The dynamic nature of trust in HRI has been underscored by many scholars (Stuck and Rogers, 2017; Rhim et al., 2023; Lewis et al., 2018). These researchers emphasize the need for viewing trust as a dynamic and evolving state rather than a static condition in HRI (Stuck and Rogers, 2017; Rhim et al., 2023; Lewis et al., 2018). This dynamic nature of trust is important in understanding the initiation and maintenance of interactions with robots over time. Therefore, considering the dynamic and context-dependent nature of trust is vital in the design and implementation of robots. Based on the multifaceted nature of trust in HRI, Park (2020) divide the idea of trust in HRI into two categories: performance-based trust and relation based trust. Performance-based trust mainly emphasises on reliability, capability, and competency of the robot at a given task, without demanding to be monitored by a human supervisor. On the other hand, relation-based trust implies the acceptance of a robot as a trusted social agent. Similarly a meta-analysis was performed to examine the factors that influence trust in HRI (Hancock et al., 2011). This human-robot trust model considered multiple factors that impacted trust, grouped in three main categories *human factors, robot factors* and *contextual factors*. *Human factors* are factors related to how users’ characteristics and abilities may impact trust (e.g., gender, age, personality traits, expertise, etc.) *Robot factors* are factors related to the robots’ performance and attributes, including adaptability, appearance, reliability, failure rate, etc. Finally, *contextual factors* include team collaborations (such as culture, communication, in-group membership, etc.) and tasking (such as task type, complexity, physical environment, etc.).

##### 2.2.2.2 Human and environmental influences on trust

From Hancock et al. (2011) model, it is evident that people’s trust in robots depend on multiple factors including: who is using the robot? what it is being used for? what is the operational context or environment? On the other hand, according to Lewin (1936), trust depends on two types of factors: *person related factors* and *environment related factors*. *Person related factors* include broader characteristics, preferences, and psychological aspects of an individual that may influence their trust in technology such as personality traits, cultural background, past experiences, health, age, and other individual factors that shape the overall perspective and behavior of a human being. *Environmental related factors* include all external factors and conditions that exert influence on an individual. According to Lewin, the environment plays an important role in shaping how individuals behave. This includes both the physical and social surroundings that individuals are in, as well as how they perceive and interpret their environment. The theory emphasizes that human behavior is not only determined by the individual, but also by the context in which the behavior takes place. The specific context in which the robot operates also contributes to the environment. For example, a medical robot in a healthcare setting may have different trust dynamics than a robot used for entertainment or household tasks.

##### 2.2.2.3 Robot characteristics and contextual variability

Considering Hancock et al. (2011) and Lewin (1936), is clear that in the context of HRI, trust depends not only on human and environment related factors but also on robot related factors. The characteristics and capabilities of the robot, such as its appearance, communication style, and intended functions, contribute to the overall trust. Different types of robots, including social robots, industrial robots, or assistive robots, can evoke varied responses and levels of trust from individuals. The functionality and performance of the robot are significant factors. If a robot makes mistakes or exhibits unreliable behavior, it can adversely affect the trust that individuals place in the technology. Compared to younger people, trust in robots may be particularly important for older adults, especially if they have any age related disability and require physical or emotional support, which can be provided by assistive robots (Giorgi et al., 2022). In this case, robots can be used to provide the required support (e.g., picking up and delivering medicines and equipment, patient monitoring, cleaning dishes, cleaning the room/house, playing games, entertain, etc.) (Bardaro et al., 2022).

##### 2.2.2.4 Trust challenges of older adults

For older adults aged 65 and over, trust is a particularly essential component of any interaction they are involved in (Stuck and Rogers, 2017), including with robots (Schwaninger, 2020). However, what constitutes trust in a robot for an older adult can be very challenging to grasp in practice. Research suggests that older adults are more likely to use a language of distrust to refer to the development of technology in society as a whole (Knowles and Hanson, 2018). The possible reasons for this can be an overestimation of a robot’s capabilities by older adults or a lack of technological readiness to implement desired functionalities (Vincze et al., 2016). However, the lack of participation of older adults at early stages of the design and development process of robots it might contribute to a language of distrust (Frennert et al., 2013). Such distrust can directly influence acceptance, as perceived shortcomings or unmet expectations may prevent older adults from integrating robots into their daily lives, even when functional benefits are evident. Conversely, widespread acceptance is far more likely when older adults have a strong sense of trust in the robot (Sawik et al., 2023). Therefore, methods need to be developed to carefully consider the multiple facets of trust to allow an exploration of the topic from the end users’ (such as older adults) perspective.

## 3 Systematic literature review methodology

In this section, we present the methodology and search strategy that were used to conduct the literature review.

### 3.1 Methodology

**FIGURE 1:**
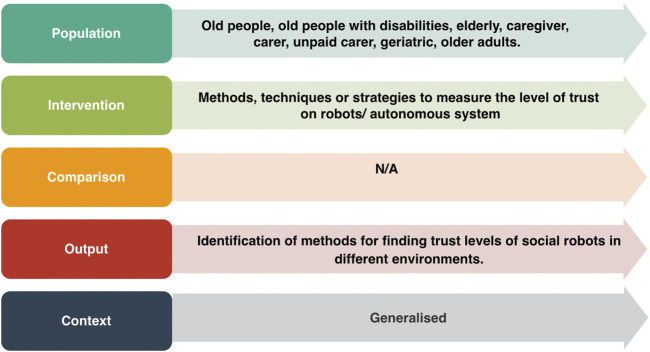
Research questions structured by the PICOC criteria.

In this work, we used the Kitchenham and Charters’ Systematic Literature Review (SLR) methodology (Kitchenham and Charters, 2007) in which the research questions and search strategy are defined first. The research questions were structured using the Population, Intervention, Comparison, Outcome, and Context (PICOC) method (Eriksen and Frandsen, 2018), as shown in Figure 1.

We were particularly interested in reviewing the methodologies used in the literature for exploring trust in SARs with older people. Therefore, our *population* consisted of *old people*, *old people with disabilities*, *elderly*, *caregiver*, *carer*, *unpaid carer*, *geriatric*, and *older adults* (the inclusion of caregivers, carers and unpaid carers as keywords was motivated by the aim of ensuring comprehensive coverage of relevant literature associated with older adults). By incorporating these additional keywords, we aimed to capture any papers that might be directly or indirectly related to older adults and their trust in SARs). Similarly, as an *intervention*, we looked at the methods, techniques, or strategies which measured the level of trust in SARs. As, we were interested in determining the methodologies, rather than comparing them, the *comparison* criteria was excluded. Our *output*, included the identification of methods for finding trust levels in SARs. Finally, we used a generalized *context* as we did not want to restrict our research questions to any particular context.

### 3.2 Search strategy

Our search strategy comprised of three phases: *identification*, *screening*, and *finalization*.

#### 3.2.1 Identification

We first searched for our keywords using three popular search engines: Scopus, IEEE Xplore, and ACM Digital Library. We thoroughly explored three databases, to ensure that no significant papers were overlooked. It’s important to note that while Scopus is comprehensive, it does not encompass all academic papers available. Therefore, our search was extensive and inclusive to minimize the possibility of missing any relevant contributions. Initially, we started the search using the keywords ‘trust’, ‘robot’, and ‘assisted living’ using the ‘AND’ operator. Our search failed as the search engines did not return any papers. Then, we broadened our search criteria and used the wildcard character ‘*’ with the keywords ‘trust’ and ‘robot’ (i.e., trust* and robot*) and removed the keyword ‘assisted living’ from our search formula. The search of the keywords trust* and robot* were limited to the abstract and the publication title. For each search engine, the search string utilized are presented in Table 1, providing transparency and facilitating the replicability of our search process. We focused on conference and journal articles from 2013–2024, since this represented the highest proportion of results, with further inclusion criteria detailed in Table 2. In brief, studies were eligible if they were in English, peer-reviewed, published after 1 January 2013, and focused on technologies measuring trust in the context of HRI, with relevance to the population criteria specified. Studies were excluded if they did not meet the language or peer-review requirements, were unavailable online, were review articles, or addressed trust in autonomous cars rather than SARs. First and the last author independently reviewed each paper to evaluate its relevance and methodological quality for inclusion in the review. Any discrepancies in assessment were resolved through discussion, ensuring that only studies meeting appropriate standards of rigor and relevance were included. For each included paper, we extracted details directly linked to our research questions, including the methodologies and metrics used to assess trust in SAR interactions RQ1; the types and categories of SARs studied and how their features influenced experimental design RQ2; the research environments and contextual factors influencing trust, with particular attention to underexplored elements RQ3; and the demographics measured, underrepresented groups, and population sizes used in the studies RQ4.

**TABLE 1:** Search Strings for each database of this literature review.

Name of search engine	Starting search string	Number of papers found	Applying filters number of papers found	Final search string
Scopus	(TITLE (trust* AND robot*) OR ABS (trust* AND robot*))	3,736	2,751	(TITLE (trust* AND robot*) OR ABS (trust* AND robot*)) AND PUBYEAR > 2012 AND PUBYEAR < 2025 AND (LIMIT-TO (LANGUAGE, “English”)) AND (LIMIT-TO (DOCTYPE, “cp”) OR LIMIT-TO (DOCTYPE, “ar”))
ACM Digital	“query”: ContentGroupTitle: (trust* AND robot*) OR Abstract: (trust* AND robot*) “filter”: ACM Content: DL	331	315	“query”: ContentGroupTitle: (trust* AND robot*) OR Abstract: (trust* AND robot*) “filter”: E-Publication Date: (01/01/2013 TO 12/31/2024), ACM Content: DL
IEEE Xplore	“Publication Title”:trust* AND “Publication Title”:robot*) OR (“Abstract”:trust* AND “Abstract”:robot*)	1,059	892	(“Publication Title”:trust* AND “Publication Title”:robot*) OR (“Abstract”:trust* AND “Abstract”:robot*) Filters Applied: Conferences Journals 2013–2024

**TABLE 2:** Inclusion and exclusion criteria for the literature search.

Inclusion criteria	Exclusion criteria
- The paper is in English	- Not in English
- Peer reviewed, obtained from journal or conference	- Not peer reviewed
- Publish on or after 1st of January 2013	- Not available online
- Focused on technologies for measuring trust in the context of HRI.	- Is a survey article (review article) or SLR.
- Papers related to population mentioned above	- Includes trust on autonomous cars
- All papers meeting the population criteria, regardless of participant’s age, involvement or use of physical robots	

#### 3.2.2 Screening

We first removed 1,119 duplicate papers from the list of 3,922 papers. To further narrow down our search within the remaining 2,803 papers, we searched for our keywords of interest for population (i.e., *old people*, *old people with disabilities*, *elderly*, *caregiver*, *carer*, *unpaid carer*, *geriatric*, and *older adults*) as defined in our PICOC criteria (see Figure 1) within the abstracts and publication titles of the 2,803 shortlisted papers. We used the ‘OR’ operator while searching for the papers that have any of these keywords. This resulted in the exclusion of 2,739 additional papers. We then assessed the remaining 64 papers for eligibility using the exclusion criteria shown in Table 2. This resulted in the exclusion of another 17 papers as they were not related to the context.

#### 3.2.3 Finalization

**FIGURE 2:**
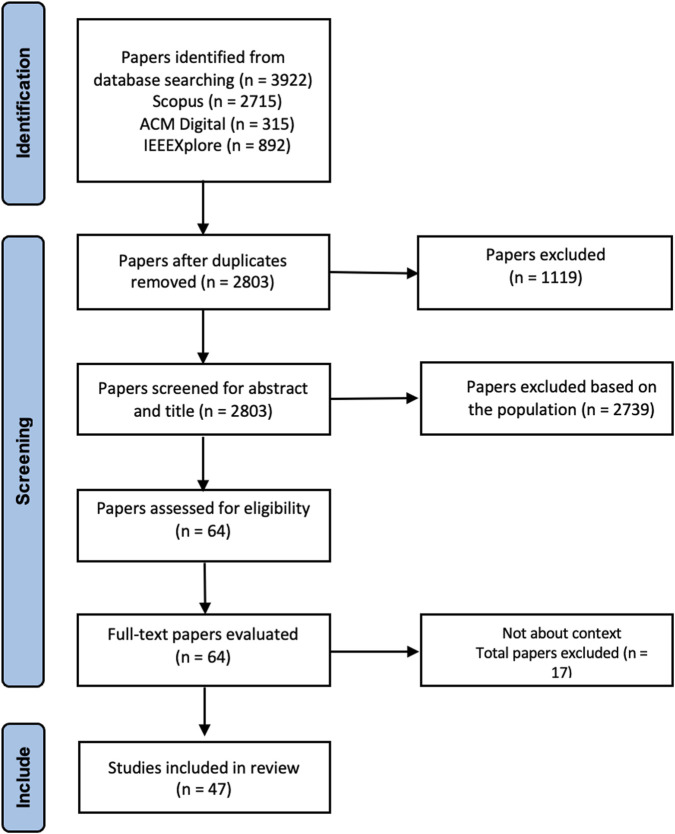
Systematic literature review flow diagram based on PRISMA flow diagram.

After completing the screening, we were left with 47 papers which were included in the review. Figure 2 shows the PRISMA flow diagram for our systematic literature review summarizing the identification, screening, and finalization phases of our methodology.

## 4 Results

In this section, we explore the extent to which our research questions are addressed from the corpus of our shortlisted 47 research papers. We first present an overview of the selected papers in Section 4.1. In Section 4.2, we present our results on how trust in SARs is measured. Section 4.3 shows results about the robots used in the studies. In Section 4.4, results related to the context in terms of research settings and factors influencing trust of the studies are presented. Finally, in Section 4.5, it is presented the demographic information with population sizes (and age ranges).

### 4.1 Overview of the selected papers

The papers included in our review were from 11 different conferences and 21 different journals. The names of these conferences and journals are listed in Table 3. Nearly 70% of these journals and conferences were multidisciplinary in nature (Medical Sciences, Technology, Social Sciences, Computer Science, Engineering, Arts and Humanities and Mathematics) and 30% were from the Computer Science category. This diverse representation underscores the collaborative and inclusive approach taken in exploring various facets of trust. The mix of these contributions gives us a wider view and shows how different areas of study collaborate to better understand the relationship between older adults and SARs. The inclusion of such a broad spectrum of disciplines contributes to a comprehensive understanding of the complex dynamics surrounding the topic of trust, shedding light on the diverse perspectives that shape our insights into the multidimensional aspects of trust in SARs. From Table 3, the most number of papers selected from any year was 8 (from 2024), while the least number of papers selected from any year was 1.

**TABLE 3:** Number of articles ( ★ = 1 article) published per year (2013–2024) across journal/conference titles.

Title name	13	14	15	16	17	18	19	20	21	22	23	24
International Conference on Universal Access in Human-Computer Interaction	★											
International Conference on Smart Homes and Health Telematics	★											
Cognitive Computation		★										
Journal of Intelligent and Robotic Systems		★										
Computers in Human Behavior			★									
Gerontechnology			★									
IEEE Industrial Electronics Society			★									
Proceedings of the Human Factors and Ergonomics Society Annual Meeting			★★									
International Symposium on Robot and Human Interactive Communication				★	★	★		★		★		★★
International Conference on Human Aspects of IT for the Aged Population					★							
Archives of Design Research					★							
International Conference on Human System Interaction						★						
Journal of Robotics						★						
Nordic Conference on Human-Computer Interaction						★						
IFIP International Conference on Human Choice and Computers						★						
SmartWorld, Ubiquitous Intelligence and Computing, Advanced and Trusted Computing, Scalable Computing and Communications, Cloud and Big Data Computing, Internet of People and Smart City Innovation							★					
Cognition, Technology and Work							★					
Journal of Medical Internet Research							★					
International Joint Conference on Computer Vision, Imaging and Computer Graphics								★				
Journal of NeuroEngineering and Rehabilitation								★				
Ergonomics									★			
Ageing and Society									★			
Informatics									★			
Telematics and Informatics									★			
International Conference on Human-Robot Interaction										★		
Sustainability										★		
Journal of Supercomputing										★		
International Journal of Social Robotics										★	★	
International Journal of Human-Computer Interaction										★		
Sensors										★	★	
Proceedings of the Future Technologies Conference											★	
International Journal of Human-Computer Studies											★	
International Conference on Intelligent User Interfaces												★
International Conference on Social Robotics												★
ACM Conference on Conversational User Interfaces												★
International conference on WorldS4												★
Journal of Open Innovation: Technology, Market, and Complexity												★
Industrial Management and Data Systems												★

**FIGURE 3:**
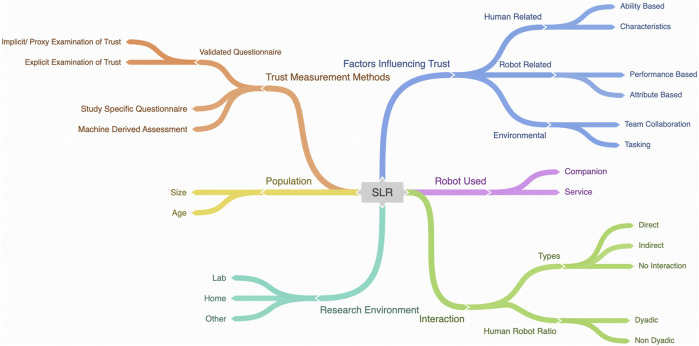
An overview of the classification of key themes found across the literature.

All of the selected papers focused on evaluating trust on SARs in the context of older adults. Therefore, the participants included in these studies were either older adults or were responsible for taking care of older adults. The nature of interactions with the robots differed across various studies. In certain studies, participants directly engaged with the robot. In contrast, in some instances, the interaction was more indirect, involving participants viewing pictures/videos of robots, while in other studies, participants relied on their perceptions of robots. Classifications used in the SLR were derived from our research questions outlined in Section 1 to capture the key themes found across the literature. Figure 3 presents an overview of our SLR classification. The complete information gathered from the studies included in our SLR is presented in the Supplementary Appendix 1 .

### 4.2 What are the methodologies and metrics used to assess trust in the interaction with SARs?

In the dynamic domain of SARs, measuring trust involves a multifaceted approach (Park, 2020). Evaluating the trust of SARs encompasses diverse methodologies, including structured questionnaires to gauge user perceptions, and qualitative insights derived from open-ended questions, interviews, and discussions. Additionally, the integration of advanced techniques like Machine Learning (ML) facilitates objective assessments of system performance. This convergence of methods creates a holistic framework for comprehensively measuring trust in the use of SARs. In our SLR encompassing 47 papers, an inquiry revolved around the methodologies employed to measure trust in SARs when interacting with older adults. The importance of answering this question lies in the critical role trust plays in the successful integration of SARs into the lives of older adults. By comprehensively examining these studies, we aimed to not only identify the diverse methods in use but also determine if there is a consensus on the most effective approach for measuring trust in this context. The significance of uncovering such consensus or trends in methodology preferences is two-fold: it informs best practices for researchers and developers, and it contributes to the establishment of standardized approaches that enhance the reliability and validity of trust measurement in SARs designed for older adults. From our thorough exploration, we identified that the studies included in the SLR employed *Validated questionnaires* that have undergone rigorous testing and validation processes, establishing their reliability and validity across various contexts; *Study specific questionnaires* tailored to the unique objectives and context of a particular research study, and machine derived trust assessment using machine learning algorithms.

#### 4.2.1 Validated questionnaires

Among the diverse methods identified for measuring trust of older adults in SARs, questionnaires emerged as a prominent tool. In this section, we provide an in-depth exploration of the specific questionnaires employed in various studies. We explore not only the names of the questionnaires but also closely examine the questions they asked. The list of questions from different questionnaires is presented in Supplementary Appendix 2. We also explored whether the questionnaires explicitly measured trust or served as proxies to assess trust in the studies. Additionally, our analysis extends to examining the strategic timing at which each questionnaire was introduced during the course of participant engagement with the SARs.

Around 51% (i.e., 24 out of 47 papers) of studies used validated questionnaires. A list of the different types of questionnaires and the papers in which these questionnaires were used, is shown in Table 4. Some questionnaires (UTAUT, Almere Technology Acceptance Questionnaire and *ad hoc* technology acceptance questionnaire) served as proxies to measure trust, while others were specifically designed to measure trust in technology and used with SARs. In addition to the names of questionnaires used in different studies, our table includes Supplementary Material denoted by specific symbols. We observed varying approaches to the timing of questionnaire introduction in the studies. Some studies introduced questionnaires at the start of the experiment, while others implemented them at the end of the interaction or experiment. Notably, the majority of studies, totaling 14, adopted an after-interaction approach, introducing questionnaires post-engagement. In contrast, only 5 studies utilized a before or start-of-experiment strategy, collecting participant feedback from the outset and 5 studies implemented a unique approach, employing the same questionnaire both before and after participant interaction. The Unified Theory of Acceptance and Use of Technology (UTAUT) questionnaire (Ahmad, 2015) and Almere Technology Acceptance Questionnaire (Heerink et al., 2010) were the most commonly used questionnaire types. Both were used in 8 out of the 20 papers which used questionnaires. Cavallo et al. (2014) used some fundamental attributes of the UTAUT Model (such as usability, attitude, anxiety, trust and quality of life). Rossi et al. (2018) adopted the version of the UTAUT questionnaire proposed by Heerink et al. (2010) (adapted and validated in the context of assistive robotics applied to elderly users). Fakhrhosseini et al. (2020) used a subset of variables from the UTAUT questionnaire which described a real-life scenario (performance adaptability, perceived enjoyment, perceived sociability, perceived usefulness, social influence, trust, anxiety, and attitude). Similarly in a study conducted by Harris and Rogers (2021), participants diagnosed with hypertension were recruited and were presented with three distinct technologies of varying complexity intended to support their health self-management. These technologies included a new blood pressure monitor, an electronic pillbox, and a multifunction robot. Subsequently, participants were interviewed, responding to a series of questions carefully crafted to assess their willingness to try these technologies. The interview questions explored a subset of the UTAUT2 model (Nordhoff et al., 2020), delving into various aspects related to the adoption and acceptance of these healthcare technologies. In a study conducted by Fitter et al. (2020), used UTAUT-inspired robot perception survey and found user trust and confidence in Baxter robot increased significantly between pre- and post-study assessments. Piasek and Wieczorowska-Tobis (2018) and Torta et al. (2014) used the Almere questionnaire that was designed specifically to assess older users’ acceptance of SAR. It was adapted from the UTAUT model in the context of assistive robots and screen agents technology proposed by Heerink et al. (2010). Lorusso et al. (2023) and Fiorini et al. (2023) used the Almere Model Questionnaire (AMQ) to assess acceptance of robot in their study, specifically for vulnerable populations such as older adults. The AMQ consisted of 10 constructs and 39 items. One of the construct was “trust” with the following item *“I would trust the robot if it gave me advice”.* In study conducted by Ishak and Nathan-Roberts (2015) used Willow Garage’s assisted living robot, and evaluated trust by applying the SEIPS 2.0 (Holden et al., 2013) framework of trust that is based on transparency, feedback, and emotion theory. In a study conducted by Huang (2022) explored factors that affect elderly customers’ acceptance and use of hotel service robots and used Technology Acceptance Model (Marangunić and Granić, 2015) that proposes two important perception factors that affect users’ technology acceptance, namely, perceived usefulness and perceived ease of use. In this study they used 6 dimensions and 19 measurement items in their questionnaire. Some researchers used multiple questionnaires in a single study (See Table 4). For example, Loghmani et al. (2019) used three metrics: observations of trust in task, self-reported trust based on the Negative Attitude Towards Robots Scale (NARS) questionnaire (Nomura et al., 2023), and a custom open-ended questionnaire. For observed trust in task, the participants’ adherence to the robot’s instructions in the time-critical task “Escape the Room” was observed and recorded as a binary value. Following the robot’s instructions indicated trust, while not following them indicated lack of trust. Self-reported trust was assessed using the NARS questionnaire, which comprised eleven items rated on a 5-point numeric response scale ranging from “I strongly disagree” (1) to “I strongly agree” (5). Participants completed the questionnaire both before and after the experiment to capture any changes in their attitude towards Pepper resulting from the experiment. A custom open-ended questionnaire was used as a manipulation check. It consisted of three yes/no questions, accompanied by an optional comment field. Similarly Wald et al. (2024) and Aharony et al. (2024) used NARS, for assessing participants’ baseline levels of anxiety towards robotic agents and Human - Computer Trust (HTC), to evaluate participants’ perception of the robot. Sorrentino et al. (2021) evaluated the usability of the robot and its services by using Cavallo et al. (2014) Ad-hoc usability/acceptability questionnaire. Study conducted by Kumar et al. (2022) used Technology Adoption Propensity (TAP) (Bittencourt et al., 2019), and NARS. Participants filled out a post-trial questionnaire measuring their perceptions of the robot (enjoyment, satisfaction, and trust). Gul et al. (2024) used two questionnaires, Propensity to Trust Scale (PTS) to investigate whether trust in robots can change even in short interaction and Trust in Automated Systems Test (TOAST) to measures trust based on two dimensions, Understanding and Performance.

**TABLE 4:** Questionnaires used to examine trust implicitly and explicitly across the literature.

Title of questionnaire	Journal/Conference paper
Implicit/Proxy examination of trust	
Unified Theory of Acceptance and Use of Technology (UTAUT) Questionnaire (Momani, 2020)	Cavallo et al. (2014) ^+^, Rossi et al. (2018) ^+^, Fakhrhosseini et al. (2020)*, Fitter et al. (2020)*^+^, Harris and Rogers (2021)*
Almere Technology Acceptance Questionnaire (ATAQ) (Heerink et al., 2010)	Torta et al. (2014) ^+^, Fracasso et al. (2022) ^+^, Piasek and Wieczorowska-Tobis (2018) ^+^, Lorusso et al. (2023) ^+^, Fiorini et al. (2023)*^+^
Ad-hoc Technology Acceptance Questionnaire	Sorrentino et al. (2021)*
Technology Acceptance Model	Huang (2022)*, Aharony et al. (2024) ^+^
Technology Adoption Propensity (TAP)	Kumar et al. (2022) ^+^
Explicit examination of trust	
Trust in Medical Technology Scale	Mann et al. (2015) ^+^
Systems Engineering Initiative for Patient Safety (SEIPS) 2.0 model (Holden et al., 2013)	Ishak and Nathan-Roberts (2015) ^+^
Human-Robot Trust Scale Questionnaire	Correia et al. (2016)*^+^, Zafrani et al. (2023) ^+^
Negative Attitude Towards Robots Scale (NARS) Questionnaire	Loghmani et al. (2019)*^+^, Kumar et al. (2022) ^+^, Wald et al. (2024)*, Aharony et al. (2024)*
Custom Open-ended Questionnaire by (Lee and Moray, 1994)	Loghmani et al. (2019) ^+^, Pak et al. (2020) ^+^
Trust Questionnaire by Jian et al. (2000)	Erebak and Turgut (2019) ^+^
Propensity to Trust Scale (PTS)	Gul et al. (2024)*^+^
Trust of Automated Systems Test (TOAST)	Gul et al. (2024)*^+^
Human-Computer Trust (HCT)	Wald et al. (2024) ^+^

This table presents information on different questionnaires used in various studies, alongside their respective study names and timings of questionnaire administration. The timings are denoted by symbols: * for introduction at the start and ^+^ for introduction after the study.

Analysing all the studies that used questionnaires, it is clear that no consensus emerged on the superiority of a particular questionnaire as well as being no consensus there is a difference in those examining trust explicitly and those examining other factors which are a perceived proxy of trust (e.g., acceptance) and a prevailing trend indicated that questionnaires were predominantly introduced after interaction.

#### 4.2.2 Study specific questions

The second most frequently employed method in our analysis was the utilization of study specific survey questions/questionnaires, with a total of 18 out of 47 studies incorporating this approach. Survey questions are inquiries presented to individuals to gather specific information, opinions, or feedback. They can be categorized into various types, including open-ended questions, closed-ended questions. Open-ended questions allow respondents to answer in their own words, providing detailed and unrestricted responses. On the other hand, closed-ended questions offer a set of pre-defined response options, such as multiple-choice answers, ‘yes’ or ‘no’ options, or rating scales (Simpson and McDowell, 2019). The study conducted by Lee et al. (2017) used a survey with open-ended questions to measure the level of trust of medical staff on robotic telepresence for medical environment. A study conducted by Poulsen et al. (2018) used open ended questions like *“Would you trust your care robot’s decision in this scenario?”*. Similarly Pascher et al. (2022) used eleven open-ended questions related to different topics like *Status quo and acceptance of technology support*, *appearance and implications*, *trust and understanding*. On the other hand, Ting et al. (2017) asked an open ended question *“How can the robot inspire trust in older adults and clinicians?”*. Hoppe et al. (2022) categorized questions in three topic, i.e., *Institutional trust (Trust in healthcare systems, Trust in regulation), Progressive trust (Trust in technology), Dispositional trust (Personality Traits)*. Stuck and Rogers (2017) defined questions based on two activities of daily living (bathing and transferring) and two instrumental activities of daily living and participants were asked in general *what a robot care provider would need to be like for them to trust it with that task and what would cause them to not trust the robot* and study conducted by Gul et al. (2024) asked an open ended question about changes in robot’s behaviour in terms of interac-tion can help them in having more trust on robot. Similarly a study conducted by Wonseok et al. (2021) measured the trust scale using a three-item scale adopted from Johnson and Grayson (2005) and used question *“I trust this umpire call”* and for measuring trust in new technology, used a question *“I usually trust a technology until it gives me a reason not to trust it”*. Aly et al. (2024) used 3 trust dimensions outlined by McKnight et al. (2002) i.e., perceived competence, benevolence, and integrity. In a study conducted by Ejdys (2022) the following question was used *“I would be able to trust the indicated technology”* and measured trust using a seven-point likert scale to evaluate how a respondent agreed or disagreed with the technology (1 = totally disagree; 7 = totally agree). Two questions using 7 scale likert (1 indicated strong disagreement and 7 indicated strong agreement) were used by Giorgi et al. (2023). The first question was *“I would TRUST a robot if it gave me advice about health supplements/vitamins”* and the second question was *“I would TRUST a robot if it gave me advice on my overall medication plan (including medication for severe illness)”*. In a study conducted by Begum et al. (2015) used quantitative formula to access trust based on behaviour coding. The coding was based on interaction of the participants with the robot and interview. Some researchers have not provided any title for their questionnaires and some used proxy questions to measure trust. For example, a study conducted by Yan et al. (2013) assessed trust by using implicit or proxy questions like *Do you feel uncomfortable when using the robot?*, *Would you be afraid of your elderly family members making mistakes or breaking something on the robot?* and used a 9-point Likert Scale (Joshi et al., 2015) with *1* indicating a *strongly negative* and *9* indicating a *strongly positive* response. According to Marin et al. Marin and Lee (2013), the perception of anthropomorphism, intelligence, safety, and likeability among older adults is influenced by the degree of aging cues exhibited by embodied agents. Aging cues refer to the visual features associated with the age of these agents. The way in which older adults perceive the aging cues of avatars can impact their expectations and trust in assistant robots and used two variables, i.e., *Unkind/kind* and *awful/nice* for assessing likability. Similarly, Mo et al. (2017) used Guo, Tan, and Cheung’s questionnaire (Madsen and Gregor, 2000) for finding trust in SARs. Branyon and Pak (2015) designed a study where trust will be measured by asking the participants a questions *“how much they trusted the robot portrayed in the vignette”* and planned to record response using on a Likert scale from 1 (not at all) to 7 (very much). Tan et al. (2024) used trust questions based on studies conducted by Jang et al. (2016) who identified trust as an individual’s confidence level in a technology and Gefen et al. (2003), who defined trust as individual’s trust in a technology can significantly increase their intention to use it in the future. Similarly Rahman (2023) used study related questions, i.e.,*“What* is your level of trust in the virtual human based on her assistance in finding the missing object?” and used a Likert scale between 1 and 5, where 1 indicates the lowest trust and 5 indicates the highest trust.), and also gave a list the tentative factors (e.g., functions, attributes, configurations, etc. of the virtual human) that can influence trust in the virtual human and assessed factors using a Likert scale between 1 and 5.

The examination of survey questions (open-ended and closed-ended questions), across different studies provides insight into the diverse approaches employed to measure trust in SARs. Each study presented unique perspectives, from the open-ended inquiries on medical staff trust in robotic telepresence to the categorization of questions based on institutional, progressive, and dispositional trust. In terms of closed ended questions by utilization of Likert scale questions, ranging from technological trust to specific scenarios like health advice, showcases the versatility of these methods. In summary, looking at how different studies ask questions helps us understand trust in SARs better. The various viewpoints and ways of asking questions show that figuring out trust in these robots is quite complex. As we keep learning, these findings help us better grasp how people trust robots, making progress in how we use them in different areas.

#### 4.2.3 Machine derived trust assessment

The third method employed by researchers involved the application of machine learning (ML), specifically reinforcement learning. Ono et al. (2015) proposed a relational trust model based on Reinforcement Learning (RL) (Shweta Bhutt, 2018). RL is a type of ML technique that enables an agent to learn in an interactive environment by trial and error using feedback from its own actions and experiences or, in other words, it is a method based on rewarding desired behaviors and/or punishing undesired ones (Shweta Bhutt, 2018). Ono et al. (2015) used the idea that in human-robot communication, RL can be used to extract features of human behaviour patterns based on trust levels on the robot (Ono et al., 2015). They played a *Give-Some Dilemma* game (Van Dijk and Wilke, 2000) and measured trust against actual behavior, expectations for cooperation, and impression evaluation. Similarly (Zhang et al., 2022), also adopted an ML-based model called sensor data-based sliding window trust model. They proposed a hierarchical implicit authentication system by joint built-in sensors and trust evaluation (Zhang et al., 2022).

From 47 studies only 2 studies used ML for measuring trust of older adults in SARs. ML is not widely used for measuring trust due to several reasons (like multifaceted concept of trust in HRI or limited availability of trust - related data). While ML has been applied to evaluate trust directly, the literature still lacks a comprehensive review on this topic (Wang et al., 2020).

Our analysis reveals a predominant utilization of validated questionnaires, with approximately 51% (24 out of 47 papers) of researchers relying on this method for measuring trust in SARs. Results indicate a predominant trend where questionnaires were introduced after participants had already interacted with the robots, whether through direct interaction or indirect interaction. In the majority of studies, it appears that researchers opted to collect participant feedback and perceptions after the exposure to robotic entities. This approach allows for a post-experience evaluation, capturing participants’ reflections and insights following their interactions with the robots. Interestingly, there was a notable exception in studies Correia et al. (2016), Loghmani et al. (2019), Fitter et al. (2020), Fiorini et al. (2023), and Gul et al. (2024), where the same questionnaire was used before and after the participants’ engagement with the robots. This unique approach provides a valuable opportunity to observe changes in perception and trust over the course of the interaction as Loghmani et al. (2019) mentioned that they wanted to determine if the attitude towards Pepper changed due to the experiment. By employing the same questionnaire before and after the interaction, researchers can identify shifts in participants’ attitudes and feelings, offering a dynamic perspective on how the robotic experience influences their perceptions. The second most frequently employed method in our SLR was the utilization of open-ended questions and interviews, with a total of 14 studies incorporating this approach. As we navigate through the specific questions used in each study, we gained a deeper understanding of the multifaceted nature of trust assessment in the context of older adults and SARs. Only two studies incorporated machine learning. It is noteworthy that no clear consensus emerged regarding the most effective method. This lack of unanimity underscores the complexity of trust measurement in SARs and suggests the need for further research and collaboration to establish standardized approaches in this evolving field.

### 4.3 What types and categories of SARs are studied in trust research studies, and how do their features shape experimental design?

In our second question, we looked at different robots used in studies about trust in SARs. We wanted to understand what types of robots researchers used to study, how older adults trust and interact with them. This exploration gave us insights into the technology used, showing us different applications that play a role in building trust between older adults and their robotic companions. We found that different robots have been used in studies and categorized them into three types based on interaction/exposure with the participants, i.e., *Direct interaction (robots)*, *indirect interaction (computer simulators, pictures, or videos of robots)* or *no interaction (no robots)*. A similar categorization was used by Naneva et al. (2020).

Among the 47 selected research papers, almost 49% (23 out of 47) utilized *robots* as part of their study. In contrast, almost 28% (13 out of 47) of the studies used *computer simulators, pictures, or videos of robots*, while in the remaining 23% (11 studies), *no robots* were used (in these studies, the perception of trust in robots was assessed through qualitative approaches, such as conducting interviews or administering questionnaires). Next, we discuss the studies in which each type of robot interaction was used.

#### 4.3.1 Direct interaction

Exploring further on studies using direct interaction, we found that 23 studies used a variety of robots. These robots were characterized by a number of different features. For example, some provided a tablet interface for interaction, and some had strong arms and hands which could be used to assist older adults in getting up or moving around. Similarly, some robots had a human-like appearance (humanoid robots) to offer a more natural interface for interaction.

We identified some key differences between the robots used in these studies conducted to find the level of trust of older adults in SARs. These differences were based on the type of robot (service or companion), whether or not they offered visual/auditory interaction, whether the robot moved around the space of study, whether or not they played games with the participants, and whether or not they performed any specific tasks like medication administration, etc.). The robots used in our selected list of studies are shown in Table 5. The following 14 studies have used companion robots in their experiments: Yan et al. (2013), Torta et al. (2014), Ono et al. (2015), Begum et al. (2015), Mann et al. (2015), Correia et al. (2016), Loghmani et al. (2019), Rossi et al. (2018), Sorrentino et al. (2021), Giorgi et al. (2023), Fiorini et al. (2023), Rahman (2023), Aharony et al. (2024), and Gul et al. (2024). Companion robots are mainly aimed at providing companionship to older adults and young children (Ruggiero et al., 2022).

**TABLE 5:** A comparison of different robots used in studies having direct interaction and the context in which they were used (Used for: I = Interaction, M = Movement, P = Playing game, D = Doing tasks).

Study	Name	Type	I	M	P	D
Yan et al. (2013)	Robot with tablet display	Companion	✓			
Torta et al. (2014) ${\;}^{•}$ ; Giorgi et al. (2023) ^-^; Rahman (2023) ${\;}^{★}$	Nao	Companion	${✓}^{•}\;$ ^-^	${✓}^{★}$	${✓}^{•}$	${✓}^{★}$
Begum et al. (2015)	ED	Companion	✓	✓		
Mann et al. (2015)	iRobi	Companion	✓			
Rossi et al. (2018) ^-^; Loghmani et al. (2019)*; Gul et al. (2024) ${\;}^{⋄}$	Pepper	Companion	${✓}^{+\;⋄}$ ^-^	✓ *	✓ *	
Sorrentino et al. (2021)	Astro	Companion	✓			
Ono et al. (2015)	PALRO	Companion	✓		✓	
Correia et al. (2016)	EMYS robot	Companion			✓	
Fiorini et al. (2023)	Ohmni robot	Companion	✓			
Aharony et al. (2024)	Gymmy Robot	Companion	✓			✓
Ting et al. (2017)	CLARC	Service	✓			✓
Cavallo et al. (2014)	Oro, Coro and Doro	Service	✓			✓
Piasek and Wieczorowska-Tobis (2018)	Tiago	Service	✓		✓	✓
Marin and Lee (2013)	Homemate	Service	✓	✓		
Ishak and Nathan-Roberts (2015)	Willow Garage’s PR2 robot	Service				✓
Newaz and Saplacan (2018)	Bot vac, Roomba and PowerBot	Service				✓
Fitter et al. (2020)	Baxter	Service			✓	
Kumar et al. (2022)	Dobot magician robot	Service				✓
Wald et al. (2024)	Obi and Stretch RE1	Service				✓

For which auditory and visual features can be useful (Lu et al., 2021). Therefore, the following studies used companion robots with auditory or visual interaction features: (Yan et al., 2013; Torta et al., 2014; Begum et al., 2015; Mann et al., 2015; Rossi et al., 2018; Sorrentino et al., 2021; Gul et al., 2024). For example, Yan et al. (2013), used an immobile companion robot which consisted of pan-tilt actuation unit, auditory, visual sensors, and a tablet display. The only interaction provided by the robot was through the movement of its display unit towards the direction of user’s voice and by tracking the user’s face once it was in view of the robot’s camera. Robot used by Begum et al. (2015) gave instructions to dementia patients on how to make a cup of tea and also got involved in social conversation. Torta et al. (2014) conducted a scenario based experiment by using a companion robot that interacted with the participants about weather, measured their blood oxygen, environmental condition, played music, showed physical exercise steps and also made out going video call and Giorgi et al. (2023) conducted an interactive experiment between elder participants and a humanoid robot Nao, where the robot provided either information-type advice or recommendation-type advice on non-prescription medicines (vitamins and over-the-counter supplements). Similarly, engagement is an important element of companionship and games can be used as a tool to keep people engaged. Therefore, the following studies used companion robots capable of playing games with humans: Ono et al. (2015), Correia et al. (2016), and Loghmani et al. (2019). For example, in Ono et al. (2015),“Give-Some Game” was played with the companion robot to find the trust on robot using RL by extracting features of human behaviour. Similarly, in Loghmani et al. (2019), the robot played “Scavenger Hunt” and “Escape the Room” games in a laboratory setting.

On the other hand, service robots were used in the following 9 studies: Ting et al. (2017), Piasek and Wieczorowska-Tobis (2018), Marin and Lee (2013), Cavallo et al. (2014), Ishak and Nathan-Roberts (2015), Newaz and Saplacan (2018), Branyon and Pak (2015), Poulsen et al. (2018), Pak et al. (2020), Fitter et al. (2020), Harris and Rogers (2021), Kumar et al. (2022), and Wald et al. (2024). Service robots are mainly aimed at assisting humans in completing tasks (Ha et al., 2022). Auditory and visual features are typically used to receive and respond to instructions for assistance. Therefore, the following studies used service robots with auditory or visual features: (Marin and Lee, 2013; Cavallo et al., 2014; Ting et al., 2017; Piasek and Wieczorowska-Tobis, 2018). For example, Piasek and Wieczorowska-Tobis (2018) used a service robot which could provide tips for healthy living and set up reminders using visual/auditory features, play 9 cognitive games (digit cancellation, letter cancellation, puzzles, hangman, memory game, Stroop test, addition of integer and decimal numbers, sorting game), and performed physical exercises. Service robots are often required to be able to perform specific tasks such as playing games, medication administration. Therefore, the following studies used service robots which could perform specific tasks: (Ting et al., 2017; Cavallo et al., 2014; Ishak and Nathan-Roberts, 2015; Newaz and Saplacan, 2018; Pascher et al., 2022; Kumar et al., 2022). For example, Ting et al. (2017) used a service robot which could perform comprehensive geriatric assessments while Ishak and Nathan-Roberts (2015) used a service robot which could administer medication. Similarly, Wald et al. (2024) used Obi for feeding and Stretch RE1 for bathing. The aim of the study was to observe trust when robot occasionally make intentional mistakes while performing two tasks, i.e., feeding and bathing.

#### 4.3.2 Indirect interaction

Exploring further on studies using indirect interaction, we found that 13 studies used computer simulator, pictures or videos of robot. For example, some videos featured robot doing exercise and in some studies they showed pictures of robot like AIIA, Baxter and HRP-4C. The following ten articles used computer simulators, pictures, or videos of robots in their studies: Aly et al. (2024), Poulsen et al. (2018), Lee et al. (2017), Mo et al. (2017), Erebak and Turgut (2019), Pak et al. (2020), Fakhrhosseini et al. (2020), Harris and Rogers (2021), Do et al. (2021), Wonseok et al. (2021), Zafrani et al. (2023), Lorusso et al. (2023), and Aly et al. (2024). A study conducted by Zafrani et al. (2023) used a video of Gymmy (a robotic system for physical and cognitive training). Similarly, studies conducted by Lee et al. (2017), Pak et al. (2020) and Erebak and Turgut (2019) used pictures of robots. Mo et al. (2017) used simulated service robot.

#### 4.3.3 No interaction

In the studies Hoppe et al. (2022), Stuck and Rogers (2018), Daniele et al. (2019), Fracasso et al. (2022), Zhang et al. (2022), Camilleri et al. (2022), Ejdys (2022), Huang (2022), Amin et al. (2024), Tan et al. (2024), and Branyon and Pak (2015), no physical robots were utilized. Instead, the perception of trust was assessed through qualitative methods, such as conducting interviews and utilizing questionnaires or a trust model using ML was proposed.

From the examination of 47 select papers we found that only 49% of these studies (23 out of 47) chose to incorporate robot as integral components of their investigations. This hands-on approach, utilizing physical robotic entities, offers a direct exploration of HRI dynamics. In a distinctive contrast, 28% of the studies (13 out of 47) opted for alternative methods by employing computer simulators, pictures, or videos of robots. This choice, which may stem from practical considerations or experimental flexibility, showcases the versatility in approaches to studying trust in the context of robotic technology. Interestingly, the remaining 20% of the studies (13 out of 47) pursued a different avenue by excluding the use of physical robots altogether. In these instances, trust perceptions were assessed through qualitative methodologies such as interviews or questionnaires. This qualitative approach allowed for a deeper understanding of trust dynamics without the direct presence of robotic entities. In examining the diverse landscape of HRI, it becomes evident that engagement plays a pivotal role in shaping the trust dynamics between older adults and SARs. The studies reviewed reveal a rich spectrum of direct interaction scenarios, where companion and service robots exhibit unique features to engage users. Companion robots, designed for companionship, using auditory and visual interaction features, enhancing engagement through conversations, games, and interactive scenarios. Service robots, focused on assisting with tasks, employ visual and auditory features for instructions and provide engagement through specific functionalities like cognitive games and physical exercises. Furthermore, indirect interaction studies using simulators, pictures, or videos showcase alternative avenues for engagement. Notably, the absence of physical robots in some studies underscores the importance of exploring trust perceptions even without direct interaction. As we navigate the evolving landscape of human-robot engagement, these findings not only contribute to understanding trust but also provide valuable insights into tailoring robotic interactions to enhance user engagement and foster meaningful connections in various contexts.

### 4.4 What are the research environments and factors influencing trust in SARs and which factors have been under explored?

In our exploration of the third question, we aimed to look into the contextual dimension of the studies in terms of research setting, the ratio of humans to robots in these research settings as well as factors influencing trust that the researchers explored. We sought to understand the settings in which these studies were conducted, specifically differentiating between laboratory (lab) and wild (e.g., home environments). The choice between these environments holds significant implications for the validity and applicability of the findings, especially given the unique needs and behaviors of older individuals (Molina-Mula et al., 2020). As laboratory setting provides controlled conditions, enabling precise measurements and controlled variables. While this offers experimental rigor, it may not fully capture the real-world intricacies and challenges that older adults might encounter when interacting with robots in their homes.

In addition to investigating the research environments, we also analyzed the types of interaction ratios commonly used in these studies. To structure this analysis, we adapted the interaction framework proposed by Sørensen et al. (2014), originally developed for human-artifact interactions. This framework categorizes interactions into four basic structures: 1. many users interacting with many artifacts, 2. one user interacting with many artifacts, 3. many users interacting with one artifact, and 4. one user interacting with one artifact. While Sørensen et al. (2014) applied this framework to digital artifacts, we tailored it to human-robot interactions, categorizing them as dyadic (1:1) or non-dyadic scenarios. Non-dyadic interactions include 1:Many (a robot engaging with multiple humans), Many:1 (multiple robots assisting a single human), and Many:Many (group-based interactions involving multiple humans and robots). This allowed us to assess which interaction ratios were most commonly employed in laboratory and in-the-wild studies, providing a clearer understanding of how SARs are typically evaluated across different contexts.

In terms of factors influencing trust, encompassed a comprehensive examination of whether the focus of trust assessment was directed towards the robot itself, the human involved, or the environmental aspects surrounding the interaction. For instance, did studies predominantly measure trust in the robot’s capabilities, reliability, and behavior? Or did the assessment pivot towards the human factors, considering aspects such as user expectations, perceptions, and preferences? Furthermore, we explored whether environmental factors, such as the physical surroundings and contextual scenarios, played a pivotal role in shaping trust dynamics.

Our selected studies were conducted in various environments such as lab, home, care homes or nursing homes. Table 6 shows the distribution of the contexts with respect to their interaction type. Lab environment was the most common context in which these studies were conducted as it accounted for almost 49% of the studies. The interaction with the robot in lab was either direct or indirect. The following studies were conducted in lab environment that have direct interaction with the robot: Yan et al. (2013), Cavallo et al. (2014), Ono et al. (2015), Mann et al. (2015), Correia et al. (2016), Ting et al. (2017), Piasek and Wieczorowska-Tobis (2018), Newaz and Saplacan (2018), Rossi et al. (2018), Loghmani et al. (2019), Fitter et al. (2020), Kumar et al. (2022), Giorgi et al. (2023), Rahman (2023), Lorusso et al. (2023), Gul et al. (2024), Wald et al. (2024), and Aharony et al. (2024) and the following 5 studies were conducted in lab but interaction was indirect: Fakhrhosseini et al. (2020), Fracasso et al. (2022), Zafrani et al. (2023), Rahman (2023), and Aly et al. (2024). The “Other” category in Table 6 includes contexts such as a library, a quiet space, and an office. The following studies were conducted in a home environment: Torta et al. (2014), Begum et al. (2015), Piasek and Wieczorowska-Tobis (2018), Sorrentino et al. (2021), and Fiorini et al. (2023). Two studies (Pascher et al., 2022; Harris and Rogers, 2021) were conducted remotely via telephone. Ejdys (2022) used Computer-Assisted Web Interview (CAWI) survey technique while the study conducted by Marin and Lee (2013) and Mo et al. (2017) was conducted in a controlled environment (i.e., a space in one of the coffee areas in an elderly center). Three studies (Correia et al., 2016; Piasek and Wieczorowska-Tobis, 2018; Harris and Rogers, 2021) used more than one context for conducting their experiments (i.e., experiments were partly conducted in a lab and partly conducted in a home environment or online via email) while the study conducted by Giorgi et al. (2023) in a lab environment called *Robot Home*, was designed to resemble a real living room/home. An experiment conducted by Cavallo et al. (2014) used three service robots in three different contexts, naming the contexts as domestic (DomoCasa Lab, a domotic house developed and managed by the BioRobotics Institute of Scuola Superiore Sant’Anna in Peccioli (Italy)), condominium (common areas, such as the entrance hall, corridors and elevator, of the building where the DomoCasa Lab is located) and urban (the surrounding outdoor pedestrian area) (Cavallo et al., 2014). A study conducted by Huang (2022), interviewees were surveyed in the bustling commercial street in Zhanjiang City in western Guangdong Province, southern China.

**TABLE 6:** Categorization of research settings in various studies based on interaction.

Interaction type	Lab	Home	Remotely	Others*	Not mentioned
Direct Interaction	Yan et al. (2013) ★ ; Cavallo et al. (2014) + ; Ono et al. (2015) ★ ; Mann et al. (2015) ★ ; Correia et al. (2016) ★ ; Ting et al. (2017) ★ ; Piasek and Wieczorowska-Tobis (2018) ★ ; Newaz and Saplacan (2018) ★ ; Rossi et al. (2018) ★ ; Loghmani et al. (2019) ★ ; Fitter et al. (2020) ★ ; Kumar et al. (2022) ★ ; Giorgi et al. (2023) ★ ; Rahman (2023) ★ ; Lorusso et al. (2023); Gul et al. (2024) ★ ; Wald et al. (2024) ★ ; Aharony et al. (2024) ★	Torta et al. (2014) ★ ; Begum et al. (2015) ★ ; Piasek and Wieczorowska-Tobis (2018) ★ ; Sorrentino et al. (2021) ★ ; Fiorini et al. (2023) ★		Marin and Lee (2013) ★ ; Mo et al. (2017) ★	Do et al. (2021) ★
Indirect Interaction	Fakhrhosseini et al. (2020) ⋄ ; Fracasso et al. (2022) • ; Zafrani et al. (2023) ★ ; Rahman (2023) ★ ; Aly et al. (2024) •	Harris and Rogers (2021) ★	Harris and Rogers (2021) ★ ; Pascher et al. (2022) ★	Pak et al. (2020) •	Poulsen et al. (2018) ★ ; Wonseok et al. (2021) ★
No Interaction		Erebak and Turgut (2019); Camilleri et al. (2022)	Lee et al. (2017); Hoppe et al. (2022); Ejdys (2022); Amin et al. (2024)	Daniele et al. (2019); Huang (2022)	Stuck and Rogers (2017); Tan et al. (2024)

*: Others include Office, Quiet room, Controlled Scenario, Bustling commercial street.

★ = 1:1 interaction (one robot, one human; dyadic).

+ = Many:1 (many robots, one human; non-dyadic).

• = 1:Many (one robot, many humans; non-dyadic).

⋄ = Many:Many (many robots, many humans; non-dyadic).

Upon reviewing 47 studies, a noticeable pattern emerges where the majority of studies into direct interactions with robots involving older adults were carried out in laboratory settings. Surprisingly, only five studies extended their examination to till. This discrepancy underscores a prevalent inclination toward controlled experimental conditions, likely driven by factors such as regulated variables and experimental control. The limited exploration of direct interactions with robots in home settings specifically for older adults suggests a potential gap in understanding how these interactions unfold in real-world home environments. There appears to be a pertinent need for increased research focusing on older adults and direct robot interactions within home settings to enhance the applicability of findings to their everyday lives. In terms of robot human ratio, Table 6 highlights a significant reliance on the dyadic interaction approach in existing studies, where trust is predominantly measured in 1:1 engagements between a robot and a participant. Only two studies explored the 1:Many interaction approach, primarily through videos of robots presented to participants, indicating limited exploration of non-dyadic interaction dynamics. These limited explorations of non-dyadic dynamics underscore the need for more comprehensive research on how trust operates in interactions involving more than two parties.

**FIGURE 4:**
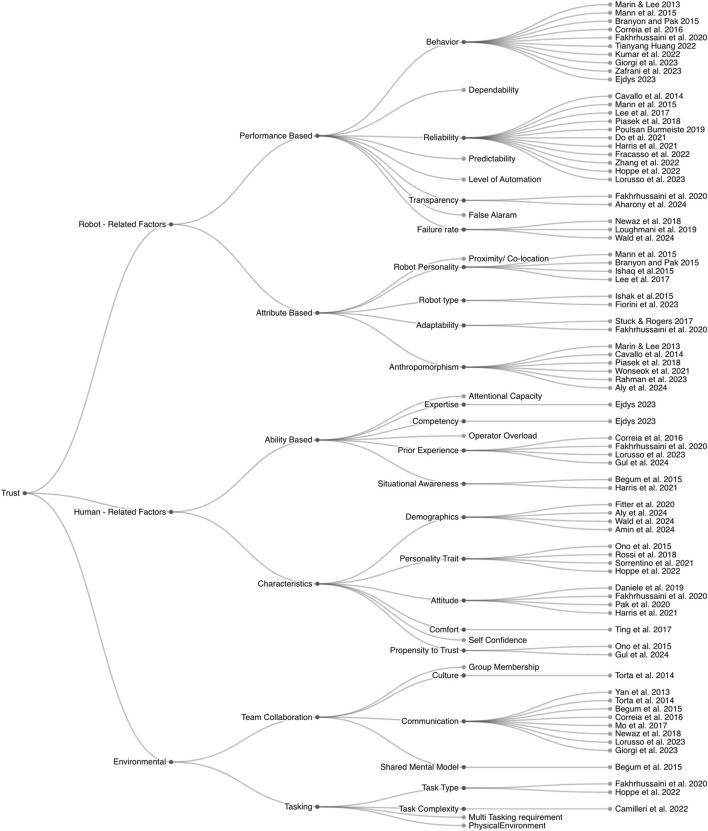
Factors influencing trust according to the revised human-trust model proposed by Hancock et al. (2021).

As part of the context, we also identified the purpose of evaluating trust within these studies. We found that studies were focused on evaluating different factors of trust. Some of the articles were focused on evaluating acceptance, with trust measurements included as part of acceptance models (i.e., Almere, UTAUT). Other articles focused on understanding specific features of robots and how that features like behaviour, reliability of the robot, etc. relates to trust. To understand better how multiple factors that impact trust have been explored within the scope of SARs and older adults, we categorised factors influencing trust according to the revised human-trust model proposed by Hancock et al. (2021), that offers a com-prehensive exploration of factors influencing trust in human-robot interactions presented in the Figure 4. Each tier of the Figure 4, from Robot - Related Factors to Human -Related Factors and Environmental, provides a breakdown of dimensions of trust in different studies. We have found that studies (Marin and Lee, 2013; Mann et al., 2015; Branyon and Pak, 2015; Correia et al., 2016; Fakhrhosseini et al., 2020; Newaz and Saplacan, 2018; Torta et al., 2014; Loghmani et al., 2019; Poulsen and Burmeister, 2019; Do et al., 2021; Erebak and Turgut, 2019; Fracasso et al., 2022; Ejdys, 2022; Huang, 2022; Kumar et al., 2022; Lorusso et al., 2023) on evaluating robot factors, particularly performance-based, including reliability, communication method, behaviour and failures. Studies Pascher et al. (2022), Branyon and Pak (2015), and Stuck and Rogers (2017) were also focused on robots’ factors, however, they explored trust from the angle of robots’ attributes that includes anthropomorphism and physical appearance. The following studies Cavallo et al. (2014), Piasek and Wieczorowska-Tobis (2018), Mann et al. (2015), and Marin and Lee (2013) explored trust according to a mix of robot factors, evaluating both aspects of performance and attributes (i.e., performance, reliability, and appearance combined).

**FIGURE 5:**
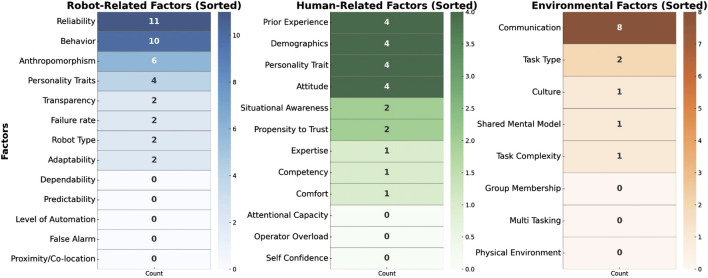
Heatmap representation of trust factors in SARs identifying key focus areas and research gaps.

Regarding trust evaluated accordingly to human - related factors, we found that studies Begum et al. (2015), Mo et al. (2017), Ting et al. (2017), Daniele et al. (2019), Pak et al. (2020), Fitter et al. (2020), Rossi et al. (2018), and Sorrentino et al. (2021) focused on characteristic-based factors such as users’ personality traits, users’ comfort with robots, attitudes towards robots and their expectancy and ability based focused on factors like situational awareness. As Environmental Factors, studies Yan et al. (2013), Zhang et al. (2022), Camilleri et al. (2022), andIshak and Nathan-Roberts (2015) were oriented to explore factors affecting trust from an angle of team collaboration, with elements such as role interdependence and interaction frequency. Finally, 5 studies Harris and Rogers (2021), Erebak and Turgut (2019), Hoppe et al. (2022), Correia et al. (2016), and Fakhrhosseini et al. (2020) did explore a combination of factors that impact trust in the scope of human - related factors, environmental factors, and robot - related factors (i.e., ability base, performance, reliability). In order to understand which contexts have been under explored, we represent factors using heatmap shown in the Figure 5. Each factor is represented along the y-axis, while the x-axis indicates the count of studies addressing that factor. The heat intensity increases with higher counts, as shown by the accompanying color bar.

Analyzing the model represented in the Figure 5, it’s evident that certain factors have been extensively explored in academic research on trust in SAR by older adults, while others may need more attention.

- In the *Robot Related Factors* category, *Reliability* under *Performance based* stands out as a heavily researched aspect, with references spanning multiple studies, including works by Cavallo et al. (2014), Piasek and Wieczorowska-Tobis (2018), and Mann et al. (2015) and many more. This indicates a consistent scholarly interest in understanding how the reliability of robots impacts the establishment of trust in SARs by older adults. The second most explored dimension is *Behavior.*
- On the other hand, *Performance based* factors like *Level of Automation*, *False Alarm*, *Predictability* and *Dependability* appear less frequently in the associated studies, suggesting that these areas are under explored or not as central to the current discourse on trust in SARs by older adults. This observation invites researchers to delve deeper into these dimensions, potentially uncovering novel insights and contributing to the broader understanding of trust dynamics.
- In *Human - Related Factors*, certain factors such as *Competency*, *Prior Experience* and *Situational Awareness* under *Ability based* have received notable attention, as indicated by references to studies by Ejdys (2022), Correia et al. (2016), Fakhrhosseini et al. (2020), and Harris and Rogers (2021) and in the *Characteristic*, *Personality trait* and *Attitude* has been explored by different studies. Conversely, factors like *Operator Overload*, *Demographics*, *Self Confidence* and *Attentional Capacity* may benefit from more extensive exploration, given their potential significance in shaping trust dynamics.
- In the *Environmental* category, *Communication* emerges as a frequently explored factor, featuring in studies by Newaz and Saplacan (2018), Torta et al. (2014), and Yan et al. (2013), and others. This aligns with the acknowledgment of the crucial role communication plays in team collaboration and overall environmental influences on trust. However, *Group Membership*, *Multi Tasking requirement* and *Physical Environment* may present opportunities for more in-depth investigations.

The analysis of the context in which studies on trust in SARs were conducted shows the significant prevalence of laboratory-based experiments. Nearly half of the studies almost 49%) were carried out in controlled laboratory environments, emphasizing the controlled conditions and precise measurements available in such settings. However, it is noteworthy that only 11% of the studies explored the home environment as a setting for their experiments. The home environment is particularly important when investigating trust among older adults, offering valuable insights into their interactions with robots in daily life (Bajones et al., 2019). This proportion indicates a potential gap in research, highlighting the need for more in-depth exploration of trust dynamics in home settings. Further investigations in this direction can provide a richer understanding of the challenges and opportunities associated with implementing SARs in real-world scenarios, especially among older populations. In our selected studies, much of the existing research predominantly relies on a dyadic interaction, where trust is measured in 1:1 engagements between a robot and a participant. In real-world applications, particularly in caregiving environments, the dynamics of trust can be significantly more complex. SARs, which show promise for use in the care of older adults, are often envisioned for scenarios involving not just the older adult, but also formal or unpaid carers. While robots may directly interact with older adults, the presence of a carer could influence how trust is established and maintained between the robot and the individual receiving care. This introduces a dynamic not typically accounted for in current research, where the carer’s role and their influence on the relationship between the older adult and the robot are not fully explored. The lack of research into non-dyadic interactions, particularly those involving multiple human actors or multiple robots, highlights a critical gap. Trust dynamics in caregiving scenarios, where a robot, carer, and older adult interact require further investigation (Gul et al., 2025). Understanding how trust functions in this triadic context is crucial, as the presence of a carer could shape both the older adult’s trust in the robot and the overall trust dynamics within the caregiving relationship. This gap in research presents an opportunity to better understand and design robots that can function effectively in multiuser caregiving environments.

Upon examining the factors influencing trust measurement methods, a clear distinction emerges between human factors and robotic factors, particularly concerning whether the measurement is subjective or objective. Upon analyzing the data regarding measurement methods and influencing factors, most studies employing subjective measurement methods predominantly focus on robotic factors, such as reliability and robot’s behavior, to gauge trust. The emphasis on these aspects of the robot’s performance suggests that subjective measurements often capture the qualitative, experiential aspects of trust influenced by the robot’s observable characteristics. Moreover, within subjective measurement methods, communication emerges as a primary environmental factor. The significance placed on communication underscores the importance of interactive and engaging features in shaping trust perceptions. These studies likely employ surveys, interviews, or observational techniques to capture the subjective experiences. Conversely, when examining into objective measurement methods, there is a shift towards human factors, specifically personality traits and propensity to trust. The utilization of these human-centric factors in objective measurements implies a more quantitative and systematic approach, likely involving computational models like machine learning to analyze patterns and behaviors objectively.

### 4.5 What demographics, underrepresented countries, and population sizes have studies measured for trust in SARs?

In this question, we directed our attention to the diverse demographics that researchers had explored in the context of measuring trust in SARs. In our investigation into population size and age range across each study, a key objective was to discern the health status of participants. Our inquiry aimed to determine whether individuals involved in these studies had age-related disabilities or if the participant pool primarily comprised healthy older adults. The demographics were characterized by analyzing the distribution of papers based on the countries to which the authors were affiliated. This approach allowed us to understand the representation of different nations in the body of literature on trust in SARs. By focusing on the author’s country of affiliation, we gained insights into the geographic diversity of research efforts, providing a lens through which to explore how trust in SARs has been studied and understood within various international contexts. Simultaneously, we sought to uncover any underrepresented demographics, thereby revealing potential gaps in our understanding. It should be noted that while our analysis identified the geographical distribution of studies, we did not perform a direct comparison of trust levels between countries, as the included studies varied widely in their contexts, aims, and trust measurement methods. Furthermore, we delved into the population sizes that featured in these studies, examining the scale at which trust in SARs had been scrutinized across different contexts.

**FIGURE 6:**
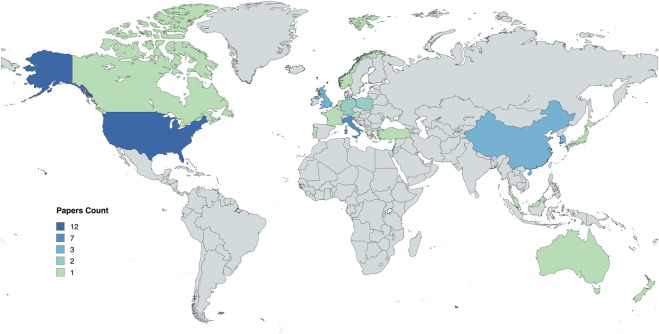
Countries of author affiliations. Colors indicate venue of publication (Grey color = no study).

The distribution of papers per country (of author affiliation) are shown in Figure 6. The map in figure reveals varying levels of research activity on SARs across different countries. The United States stands out with the highest number of papers, totaling 12. Following closely is Italy, contributing significantly with 7. Countries such as the United Kingdom, China, Germany, Israel and Korea have also shown notable research engagement, each having either 2 or 3 papers on SARs. On the other hand, numerous countries, including Australia, Austria, Canada, France, Japan, Netherlands, Malaysia, New Zealand, Norway, Portugal, Singapore, and Turkey, each have one paper. It’s noteworthy that several regions, particularly in Africa and South America, appear to be underrepresented in the provided data, as they do not have any recorded papers on trust of older adults in SARs. This indicates a potential area for future research growth and collaboration to ensure a more globally inclusive perspective SARs studies. Table 7 shows the population sample sizes used in each of our selected studies. The largest population size was 1,149 (in Ejdys (2022)) while the smallest population size was 4 (in Piasek and Wieczorowska-Tobis (2018)).

**TABLE 7:** Population statistics for studies where trust in robots was examined.

Study	Age range (years)	Mean age (years)	Sample size
Companion Robot			
Yan et al. (2013)	30–40 and ≥ 55	Not mentioned	15
Torta et al. (2014)	70–95	77	8
Begum et al. (2015)	≥ 55	77.8	10
Mann et al. (2015)	19–65	30	65
Correia et al. (2016)	Not mentioned	24.31	60
Rossi et al. (2018)	53–82	61.16	20
Fakhrhosseini et al. (2020)	86–94	90.21	22
Sorrentino et al. (2021)	72–92	83.33	7
Giorgi et al. (2023)	60–80	69.13	30
Gul et al. (2024)	18+	30	15
Rahman (2023)	Not mentioned	25.71	20
Fiorini et al. (2023)	Not mentioned	Not mentioned	11
Aharony et al. (2024)	75–85	Not mentioned	21
Service Robot			
Marin and Lee (2013)	62–91	74.58	52
Cavallo et al. (2014)	65–85	73.8 ± 6.0	35
Ting et al. (2017)	60–93	Not mentioned	24
Piasek and Wieczorowska-Tobis (2018)	≥ 65	78.5	4
Newaz and Saplacan (2018)	≤ 65	Not mentioned	11
Fitter et al. (2020)	18–36, 54–70	23.6, 59.6	20, 19
Huang (2022)	60–64, 65–69, 70–79, ≥ 80	Not mentioned	67, 67, 34, 14
Kumar et al. (2022)	67–87, 65–90	78, 72.59	32, 22
Wald et al. (2024)	Not mentioned	26.1 ± 11.5, 81.9 ± 7.6	19
Other*			
Mo et al. (2017)	Not mentioned	75.71	14
Poulsen et al. (2018)	Not mentioned	Not mentioned	102
Pak et al. (2020)	18–22, 65–79	18.7, 70.53	85
Harris and Rogers (2021)	65–84	75	23
Do et al. (2021)	60–89	73.4	30
Wonseok et al. (2021)	18–74	34.43	200
Fracasso et al. (2022)	50–64, 65–85	59.16, 72.4	197
Pascher et al. (2022)	30–69	Not mentioned	12
Lorusso et al. (2023)	≥ 60	Not mentioned	57
Zafrani et al. (2023)	65–85	71.73	384
Aly et al. (2024)	Not mentioned	Not mentioned	36(young), 27(old)
No Robot Used			
Stuck and Rogers (2017)	≥ 65	Not mentioned	15
Lee et al. (2017)	30–60	Not mentioned	14
Erebak and Turgut (2019)	19–40	30	102
Daniele et al. (2019)	65–74	Not mentioned	35
Hoppe et al. (2022)	Finland: 42–62, Germany: 26–62, Sweden: 37–56	55.2, 44.1, 44.5	20
Ejdys (2022)	≥ 40	Not mentioned	1,149
Tan et al. (2024)	35–65	Not mentioned	387

*: Other = computer simulators, pictures, or videos of robots.

Among the studies that used a service robot, the largest population size was 67 (in Huang (2022). On the other hand, among the studies that used a companion robot, the largest population size was 65 (in Mann et al. (2015)). In studies that used computer simulators pictures, or videos of robots are categorised as Other and the largest population size was 384. In our collection of 47 papers, only 7 studies did not involve any participants. For instance, the research by Zhang et al. (2022) did not use real robots and, as a result, did not include any participants in their experiments. This might be because they were focused on developing a model or method rather than testing it with people. Similarly, Camilleri et al. (2022) and Branyon and Pak (2015) created a method or model but did not deploy or test it with participants. The decision could be attributed to the early stages of development or a focus on theoretical aspects before engaging in practical testing. Additionally, in the study by Ono et al. (2015), the number of participants involved was not specified, which could be due to oversight in reporting or a deliberate omission in their research methodology. The ages ranges of the participants in each of the selected studies are shown in Table 7. 25 studies have included older adults (aged 60, or over) as their participants. The eldest person included in these studies was 95 years old (in Torta et al. (2014). The mean age in these studies ranged from 30 to 90.21 years. Six studies (Correia et al., 2016; Mo et al., 2017; Poulsen et al., 2018; Zhang et al., 2022; Camilleri et al., 2022; Ono et al., 2015) did not mention the age ranges of their population while one study (Ishak and Nathan-Roberts, 2015) did not include any participants. All the studies that used service robots included older adults as their participants. In these studies, the mean population age ranged from 73.8 years to 78.5 years. 7 out of 8 studies which used companion robots, included older adults as their participants. In these studies, the mean population age ranged from 24.31 to 90.21. In two studies (Mo et al., 2017; Correia et al., 2016), only the participants’ average age information is available while information about the age ranges of the participants is not available.

Our comprehensive analysis reveals a concentration of research efforts on SARs predominantly in the United States and Italy, with these two countries contributing significantly to the field. The map of research activity illustrates a global landscape with notable research engagement in these regions. However, it’s crucial to acknowledge the under representation of several areas, particularly in Africa and South America, pointing to potential opportunities for research growth and collaboration to ensure a more balanced and globally inclusive perspective on SARs.

Looking into participant demographics, our findings indicate a predominant focus on healthy older adults in the selected studies. This observation raises awareness of potential biases in the participant selection process and underscores the importance of expanding research to include a more diverse representation of older adults, encompassing those with varying health conditions and backgrounds. As the field of SARs continues to evolve, addressing these geographical and demographic imbalances will contribute to a more comprehensive understanding of the dynamics surrounding trust and interaction with robots, particularly among older populations.

## 5 Discussion

We conducted a detailed review of studies which were published between 2013 and 2023 to analyse and compare the conventions used as well as identify key research challenges and gaps. This SLR provides an understanding of current research in SARs and how trust has been measured. Our findings reveal six main challenges that need to be considered when measuring trust of older adults in SARs.

### 5.1 Subjective measurements of trust: reflecting on the lack of standardisation

In investigating RQ1, how trust is measured in SARs for older adults, our review exposes a lack of standardized methods for finding trust in SARs. One of the most popular methods used was questionnaires. 14 out of 47 papers, researchers used proxy examination of trust and used questionnaires like ATAQ (Eriksen and Frandsen, 2018) or and UTAUT (Ahmad, 2015). Apart from questionnaires, study specific surveys/questionnaires were also popular tools for measuring the level of trust in SARs for finding trust of older adults. Additionally, we observed a lack of consensus regarding the preferred method and its suitability for specific types of experiments. Our second finding in terms of methodology is that existing methods used for measuring trust of old people in SARs were based on subjective evaluation of trust. Subjective evaluation or self-report measures is typically based on personal assessment of the environment. Self-report measures are intrusive, however these methods are not viable in applied setting. In old age, capability to correctly assess the environment may be affected due to cognitive limitations (Erickson et al., 2022). According to Edelstein et al. (2010), the accuracy, reliability, and validity of older adult self-reports is mixed, suggesting that one should be cautious when using the self-report method and, when possible, utilize multiple methods.

Due to advancement in automation and technology, different standards are being developed (e.g., Standard for Clinical Internet of Things (IoT) Data and Device Interoperability with TIPPSS–Trust, Identity, Privacy, Protection, Safety, Security (IEEE, 2019)) have been developed to ensure consistency and interoperability across solutions and technologies. Trust in SARs needs to have some standard framework, as standard framework provide many benefits like consistency in methods, technology, terminology and work-processes. Another important consideration is the use of objective evaluation along with subjective evaluation. Due to limitations of subjective evaluation methods of trust in old age, it is important to also use objective evaluation methods for measuring trust in SARs. Given the identified gaps in standardized trust measurement methods and the limitations of subjective evaluations alone, we propose a conceptual framework for measuring trust in SARs. This framework integrates both subjective self-reports (validated questionnaires and study-specific surveys) and objective evaluation methods (behavioral and physiological measures) to provide a more reliable and holistic assessment of trust.

#### 5.1.1 A proposed conceptual framework for measuring trust

**FIGURE 7:**
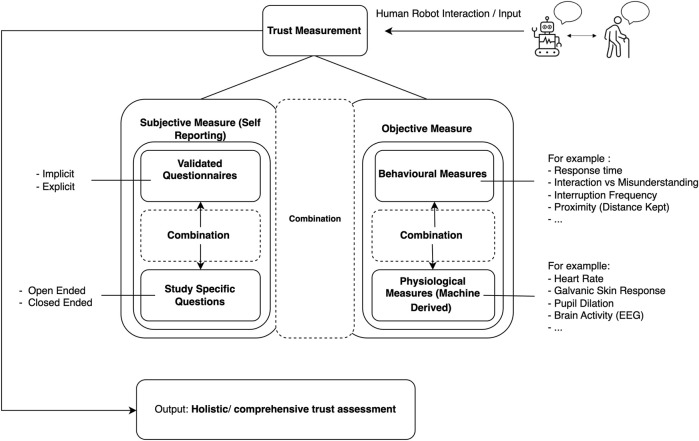
Proposed trust assessment framework Subjective Objective Trust Assessment - HRI (SOTA - HRI).

The Subjective Objective Trust Assessment HRI (SOTA - HRI) is a novel trust measurement framework developed to assess trust in older adults comprehensively and holistically. The proposed framework is inspired by the methodology outlined in the Gebhard et al. (2021), which explores theorized and empirically supported trust factors. While the referenced paper focuses on identifying trust factors in various contexts, we have used their approach to define a trust measurement framework that incorporates subjective and objective measures of trust. Our framework integrates insights from prior studies, ensuring that trust is assessed using validated constructs and empirically supported methods. Given that trust is a multidimensional construct, especially for older adults who may face unique cognitive, emotional, and social challenges, this framework adopts a dual-method approach—combining self-reported subjective measures and objective measures. The aim of the SOTA-HRI framework is to provide a reliable, holistic, and comprehensive method for measuring trust in SARs for older adults. Figure 7 represents the SOTA - HRI framework. The trust measurement process starts with the interaction between the older adult and the robot, which serves as the input for collecting data. This interaction provides the foundation for measuring trust, as it involves the exchange of information, behavioral responses, and emotional engagement during HRI (Lechevalier et al., 2025). The framework then processes this input through two parallel evaluation methods: subjective measures and objective measures, which are combined to produce a holistic and comprehensive trust assessment.

Subjective measures capture participants’ self-reported experiences of trust (Detailed explanations can be found in the Section 4.2). This involves two types of instruments: *Validated Questionnaires:* These are standardized tools used to assess trust levels across various contexts. *Study-Specific Questions:* Custom-designed surveys tailored to the specific study context.

The objective measures section on the right side of the Figure 7, captures objective data from the interaction through two key methods: *Behavioral Measures:* identified and selected based on a comprehensive review of existing literature on trust in automation specifically HRI (see Table 8). These include observable behaviors such as response time, interruption frequency, proximity (distance kept from the robot), and the quality of interactions (successful interactions vs. misunderstandings). *Physiological Measures (Machine-Derived):* These involve collecting physiological responses from the older adult using sensors and devices (See Table 9). Common metrics include heart rate (HR), galvanic skin response (GSR), pupil dilation and brain activity (EEG). These measures provide implicit indicators of trust, offering insights into the emotional and cognitive state of the older adult during the interaction.

**TABLE 8:** Behavioral Metrics in SOTA - HRI match to the literature.

Metric	Description	Outcome	Source
Response Time	Duration between participant asking a question and the robot’s response	“Our results also showed a correlation between perceived response delay and user trust, indicating that users are more likely to trust the chatbot that was perceived to respond fast.”	Zhang et al. (2024)
Successful Interactions	Number of smooth communication instances without misunderstandings	“Trust decreased significantly after Pepper began making mistakes in the solicited case compared to both the pre-corrective and post-corrective case.”	Ye et al. (2019)
Interruption Frequency	Number of times participants interrupt or take control from the robot	“Intervention is a behavioral opposite of reliance, in which participants intervene and take over control from the teammate. The act of intervening is indicative of a state of distrust that exceeds this hesitancy barrier.”	Kohn et al. (2021)
Proximity	Average distance maintained between participants and the robot	“Participants were approached by a humanoid domestic robot two times and indicated their comfort distance and trust.”	Miller et al. (2021)

**TABLE 9:** Physiological Measures In SOTA - HRI match to the literature.

Physiological measure	Description	Outcome	Reference
Heart Rate (HR)	Monitors changes in heartbeat to reflect stress or relaxation during interaction	“The findings confirmed that physiological measures such as HR and SKT are significant indicators of trust, and the use of multiple physiological behaviours collectively can enable real-time sensing of human trust in robots.”	Alzahrani and Ahmad (2024)
Galvanic Skin Response (GSR)	Tracks changes in skin conductivity caused by emotional arousal	“Long system usage time strengthens the relations between dynamic trust and the GSR, HR.”	Yi et al. (2023)
Pupil Dilation	Measures changes in pupil size, indicating attention or cognitive load during trust-related tasks	“We observed that interaction partners with dilating pupils are trusted more than partners with constricting pupils.”	Kret and De Dreu (2017)
Brain Activity (EEG)	Examines neural activity in trust-related brain regions, such as the prefrontal cortex	“The findings indicate the existence of a correlation between trust levels and the EEG data, thus offering a promising avenue for real-time trust assessment during interactions, reducing the reliance on retrospective questionnaires.”	Campagna and Rehm (2023)

At the center of the figure, the combination block represents the integration of both subjective and objective data. This integration is important because subjective evaluations alone may be influenced by biases, cognitive limitations, or emotional states, especially in older adults. By combining self-reported perceptions with externally observed and machine-derived data, the framework ensures a more balanced and reliable assessment of trust. The output of the framework, as shown at the bottom of the figure, is a holistic and comprehensive trust assessment. By combining multiple evaluation methods, the framework ensures that trust is assessed accurately, capturing both self-reported trust levels and trust-related behaviors and physiological responses.

### 5.2 Multifacet understanding of factors influencing trust, robots and context

From our analysis of the RQ2 and RQ3, where we examined 47 selected research papers, we found that approximately half (23 out of 47) utilized robots in their studies. Notably, our findings suggest a predominant use of robots in controlled environments, such as labs. This controlled setting, while providing a scenario conducive to research, may influence the level of trust observed in older adults, as they might feel more at ease with expert assistance in a lab environment, potentially impacting the generalizability to home environments where trust dynamics may differ. Trust is crucial for sustainable interaction with assistive technology, especially in sensitive contexts like homes and intimate spaces (Schwaninger, 2020). Hence, it is expected that the level of trust in SARs shown by the older adults in a lab environment may not be a true reflection of their level of trust in a home environment. Similar findings were reported in Whelan et al. (2018). It needs to be investigated further to know if the level of trust increases or decreases with time and if living in homes have a positive or a negative impact on the level of trust in SARs. Similar observations were made by (Bajones et al., 2019) who conducted field trials with a mobile service robot in a private home environment and found that trials should be moved to homes in order to better understand real world challenges. Bemelmans et al. also indicated that further investigation is required to evaluate the effects of SARs within real elderly care settings (Bemelmans et al., 2012). In examining the factors influencing trust in SARs in RQ3, the studies analyzed were categorized into three primary groups: robot-related factors, human-related factors, and environmental factors. Robot-related factors, particularly those related to performance, received substantial attention, with a focus on reliability, behavior, and the handling of failures by SARs (Marin and Lee, 2013; Mann et al., 2015; Correia et al., 2016; Fakhrhosseini et al., 2020; Branyon and Pak, 2015; Ejdys, 2022; Giorgi et al., 2023; Zafrani et al., 2023; Piasek and Wieczorowska-Tobis, 2018; Poulin and Haase, 2015; Do et al., 2021; Lee et al., 2017; Kumar et al., 2022; Wonseok et al., 2021; Huang, 2022; Fracasso et al., 2022). Similar findings were reported by Brule et al. (2014) that performance based factors have a large influence in perceived trust in HRI. Another significant subset of studies delved into the impact of robot attributes, such as anthropomorphism and physical appearance, highlighting the importance of considering emotional and psychological aspects in designing SARs for HRI, especially in the context of older adults. While some studies adopted a comprehensive perspective by considering both performance and attributes in evaluating trust, there was no consensus on the dominant factor. Human-related factors, encompassing user characteristics and traits, were explored in a separate cluster of studies, emphasizing the role of individual and psychological aspects in trust formation. Additionally, 13 studies demonstrated the influence of environmental factors, particularly those related to team collaboration, suggesting that trust is shaped not only by the robot or the individual user but also by the broader context in which SARs are utilized. This broad understanding is essential for the effective integration of SARs into the lives of older adults, fostering trust and acceptance (Langer et al., 2019). While a subset of studies recognized the interplay between human, environmental, and robot factors, acknowledging the complexity of trust formation, there was no consensus on which factor exerted greater influence. Moreover, certain factors, such as level of automation, false alarm, predictability, dependability, operator overload, demographics, self-confidence, attentional capacity, group membership, multi-tasking requirements, and physical environment were notably under explored. This highlights gaps in the current research landscape, suggesting avenues for future exploration to comprehensively understand the broad nature of trust in SARs.

From our analysis of RQ4, we examined demographics according to author affiliation in which study on SARs was conducted. The distribution of studies across these countries reveals a varying level of research activity in this field. Notably, the majority of studies have been conducted in countries with strong research and development ecosystems, including the United States and Italy, which have 8 and 6 studies, respectively. Furthermore, several other nations in Europe and Asia have contributed to the research landscape, each with one to two studies. It is essential to acknowledge the varying levels of research activity in different regions, as this may reflect disparities in technological exposure, cultural contexts, and research priorities when exploring trust within the area of SARs and older adults as different cultures have different levels of trust and effective interaction with robots. The study highlighted in Papadopoulos et al. (2018) emphasized that older adults’ acceptance of healthcare robots is shaped by individual factors such as cultural background and suggested that culturally competent assistive robots should be employed ethically, serving as valuable tools for human caregivers and that these robots are designed to complement rather than replace human caregivers, in accordance with the principles outlined in the BSI 2016 guidelines (BSI, 2024). Different demographics exhibit varying levels of trust and effectiveness in engaging with robotic technology (Seaborn et al., 2023). Recognizing and addressing these regional variations in technological familiarity is essential for achieving a more comprehensive understanding of how trust in SARs is influenced by human factors when interacting with older adults. Although our analysis did not compare trust levels across countries, prior literature indicates that such differences, such as the higher trust in robots often reported in Japan compared to many European contexts, may reflect deeper cultural orientations toward technology, historical exposure to robotics, and differing institutional frameworks (Ikari et al., 2023; Castelo and Sarvary, 2022). Future research incorporating cross-cultural designs and standardized trust measures could provide valuable insights into how these factors shape trust in SARs among older adults. We also found that participants in the selected studies were healthy older adults with no age-related disabilities. To foster a more comprehensive understanding of trust in SARs, there is a clear need for increased involvement of older adults with age-related disabilities in future studies. As age-related disabilities can impact an individual’s physical, cognitive, and social capabilities, which in turn may influence their confidence and trust in using technology (Birkhäuer et al., 2017). By intentionally including individuals facing age-related challenges, researchers can explore and develop methods specifically tailored to address the unique trust dynamics that may arise in this demographic. More involvement of old adults with age-related disabilities is required to investigate methods for finding trust in SARs.

### 5.3 Limitations and future work

There are some limitations of our work we would like to acknowledge. For example, we only included papers written in the English language and limited our research for 11 years, i.e., from 2013 until 2024, this was because it represented the largest proportion of results; we excluded articles that were not published in peer-reviewed journals or conferences, and thus we may have missed out on some research and commercial solutions as a result. Additionally, while all included studies underwent independent screening by two authors to assess their methodological appropriateness and relevance, we did not apply a formalised risk-of-bias assessment tool such as AXIS (Downes et al., 2016), MIXED METHODS APPRAISAL TOOL (MMAT) (Hong et al., 2018), or Risk of Bias (RoB) (Whiting et al., 2016). The use of such a tool could provide a quantitative appraisal of study quality and comparability, and we recommend its incorporation in future reviews to further strengthen methodological rigour. Building upon the current findings, our future work aims to undertake longitudinal studies with SARs involving older adults within their home environments to explore whether initial trust in SARs is sustained or evolves, while also providing a detailed analysis of participant demographics, including age ranges, gender balance, cultural contexts, and the inclusion of older adults with disabilities, to better understand how these factors influence interactions with SARs and influence trust. Additionally, future research could explore advanced methods for measuring trust as psychophysiological assessment, such as data-driven fuzzy logic approaches, in the broader context of HRI research.

## 6 Conclusion

An SLR was conducted with the goal of studying technologies or methods used for measuring trust in SARs and to understand the types of robots, sample populations, and the contexts of these studies. 47 articles were reviewed in depth and three methods were identified that were used to measure the level of trust of older adults in SARs. The most common method was questionnaires but with limited standardization across them in how trust was measured. The challenge with the use of questionnaires is the lack of standardization. Additionally, studies have mostly been carried out in a controlled environment (such as labs) with questions remaining on the representativeness in comparison to more natural environments (e.g., homes) and the transferability of the findings. We analyzed factors influencing trust and found no consensus on factor that exerted greater influence, this highlights exploration of factors comprehensively to understand broad nature of trust in SARs. The distribution of studies across countries reveals a varying level of research activity, with the majority of studies been conducted in countries with strong research and development ecosystems, including the United States and Italy, this may reflect disparities in technological exposure, cultural contexts, and research priorities when exploring trust in SARs and older adults. We also found limited study with older adults with disabilities or additional care needs, with studies predominantly focusing on healthy populations.

## Data Availability

The raw data supporting the conclusions of this article will be made available by the authors, without undue reservation.
