# Supplementary material for: Conventions and research challenges in considering trust with socially assistive robots for older adults
Source: Front Robot AI. 2025 Nov 26;12:1631206. doi: 10.3389/frobt.2025.1631206 (PMC12690213; doi:10.3389/frobt.2025.1631206)
Supplement: Supplementary file 1 [file DataSheet1.pdf]

Appendix

1 SUPPLEMENTARY INFORMATION FOR EACH STUDY

Table S1. Summary of methodologies, robot types, participant populations, and study contexts.

Study	Methodology	Robot(s)	Population	Context	Factor/Purpose
Yan et al. (2013)	Questionnaire	Robot with tablet display	15 (30–40 yrs)	Lab	Environmental
Marin and Lee (2013)	Questionnaire	Homemate	52 (62–91 yrs)	Controlled scenario	Robot-Related
Torta et al. (2014)	Questionnaire	Nao	8 (70–95 yrs)	Smart home	Robot-Related
Cavallo et al. (2014)	Questionnaire	Oro, Coro, Doro	35 (65–85 yrs)	Lab/surroundings	Robot-Related
Ono et al. (2015)	Reinforcement learning	PALRO	Not mentioned	Lab	Human-Related
Branyon and Pak (2015)	Video vignette survey	Baxter (video)	100 (18–23 and 65–85 yrs)	Home (via email)	Robot-Related
Ishak and Nathan-Roberts (2015)	Questionnaire	PR2	No participants	Not tested	Robot-Related
Begum et al. (2015)	Behavioral coding	Robot ED	10 (¿55 yrs)	Simulated home	Human-/Environmental
Mann et al. (2015)	Questionnaire	iRobiQ	65 (19–65 yrs)	Lab	Robot-Related
Correia et al. (2016)	Card game + questionnaire	EMYS	60 (age not mentioned)	Lab/Tournament	Multiple
Ting et al. (2017)	Interview	CLARC	20 (60–90 yrs)	Lab	Human-Related
Mo et al. (2017)	Questionnaire	Simulated robot	14 (avg. 75.71 yrs)	Office	Environmental
Stuck and Rogers (2017)	Interview	No robot	15 (¿65 yrs)	Not mentioned	Multiple
Lee et al. (2017)	Survey + interview	No robot	14 (not mentioned)	Quiet spaces	Robot-Related
Piasek and Wieczorowska-Tobis (2018)	Questionnaire	Tiago	Not mentioned	Home/Lab	Robot-Related
Rossi et al. (2018)	Questionnaire	Pepper	21 (53–82 yrs)	Lab	Human-Related
Newaz and Saplacan (2018)	Interview	Semi-autonomous devices	11 (¿65 yrs)	Lab	Robot-Related
Poulsen et al. (2018)	Open-ended questions	Med delivery robot	102 (not mentioned)	Not mentioned	Robot-Related
Loghmani et al. (2019)	Questionnaire	Pepper	9 (20–32 yrs)	Lab	Robot-Related
Daniele et al. (2019)	Interview	No robot	35 (¿65 yrs)	Quiet room	Human-Related
Erebak and Turgut (2019)	Questionnaire	AILA, HRP-4C (pictures)	102 (19–40 yrs)	Nursing home	Robot-/Human-Related
Fakhrhosseini et al. (2020)	Questionnaire	Jibo, Google Home, Alexa	22 (85–99 yrs)	Lab	Robot-/Human-Related
Pak et al. (2020)	Questionnaire	Baxter (pictures)	85 total (18–22, 65–79 yrs)	Not mentioned	Human-Related
Fitter et al. (2020)	Questionnaire	Baxter	39 total (18–36, 54–70 yrs)	Lab	Human-Related
Sorrentino et al. (2021)	Questionnaire	Astro	7 (72–92 yrs)	Residential facility	Human-Related
Harris and Rogers (2021)	Questionnaire + interview	Healthcare robot	23 (65–84 yrs)	Online + Lab	Robot-/Human-Related
Do et al. (2021)	Surveys	3D-printed robot	30 (60–90 yrs)	Not mentioned	Robot factor
Wonseok et al. (2021)	Questionnaire	Baseball robot (video)	200 (18–74 yrs)	Not mentioned	Robot-Related
Pascher et al. (2022)	Open-ended questions	Cobots	12 (30–69 yrs)	Telephone interview	Robot-Related
Hoppe et al. (2022)	Interview + questionnaire	No robot	20 (42–62 yrs)	Telephone interview	Robot-/Human-Related
Fracasso et al. (2022)	Questionnaire	No robot	197 (50–85 yrs)	Lab	Robot-Related
Zhang et al. (2022)	ML model	No robot	No participants	Lab	Robot-Related
Huang (2022)	Questionnaire	No robot	218	Commercial street	Robot-Related
Camilleri et al. (2022)	Discussion-based	No robot	No participants	Care home	Environmental
Ejdys (2022)	CAWI	No robot	1149 (¿40 yrs)	Online	Robot-/Human-Related
Kumar et al. (2022)	Questionnaire	Dobot Magician	54 (65–90 yrs)	Lab	Robot-Related
Lorusso et al. (2023)	Almere model	Ohmni (video)	57 (¿60 yrs)	Controlled environment	Multiple
Giorgi et al. (2023)	Questionnaire	Nao	30 (60–80 yrs)	Lab	Robot-/Environmental
Zafrani et al. (2023)	Trust scale	Video	384 (65–85 yrs)	Lab	Robot-Related
Aly et al. (2024)	Questionnaire	Video	62	Lab	Robot factor
Gul et al. (2024)	TOAST & PTS	Pepper	15 (20–45 yrs)	Lab	Human-Related
Tan et al. (2024)	Questionnaire	No robot used	387 (35–65 yrs)	Online	Robot-/Human-Related
Wald et al. (2024)	Questionnaires	Stretch RE1 & Obi	19 (not mentioned)	Lab	Robot-/Human-Related
Rahman (2023)	Questionnaire	Nao	20 (not mentioned)	Lab	Robot-Related
Fiorini et al. (2023)	Almere Questionnaire	Ohmni	11 (not mentioned)	Home	Robot-Related
Amin et al. (2024)	Questionnaire	No robot used	274 (¿65 yrs)	Online	Robot-Related
Aharony et al. (2024)	Questionnaire	Gymmy Robot	21 (75–85 yrs)	Old home facility	Robot-Related

2 QUESTIONS USED IN DIFFERENT STUDIES

Study	Questions
Yan et al. (2013)	Proxy Questions (9-point scale from 1 = strongly disagree to 6 = strongly agree):<br>- How often do you use video-conferencing technology?<br>- Do you think the robot is useful for elderly people?<br>- Do you think elderly people are able to quickly learn how to use the robot?<br>- Would you like to use the robot for video-conferencing with your elderly family members?<br>- Do you feel that it is convenient to use the robot for video-conferencing with your elderly family members?<br>- Do you feel uncomfortable when using the robot?<br>- Would you be afraid of your elderly family members making mistakes or breaking something on the robot?<br>- What is an acceptable price for the robot?<br>1) $>$ \$250<br>2) \$250–500<br>3) \$500–750<br>4) \$750–1000<br>5) $>$ \$1000<br>- If you could have this robot, how soon from now would you want it?<br>1) Immediately<br>2) A few months<br>3) One year<br>4) A few years<br>- Rank your preference for the following applications of the robot (1 = most preferred, 5 = least preferred):<br>1) Monitor baby<br>2) Talking with elderly<br>3) Receptionist<br>4) Security and surveillance<br>5) Business meeting
Torta et al. (2014); Rossi et al. (2018); Piasek and Wieczorowska-Tobis (2018)	- I would trust the robot if it gave me advice.<br>- I would follow the advice the robot gives me.
Cavallo et al. (2014)	- I would trust in the robot's ability to perform this task.

Continued on next page

Study	Questions
Mann et al. (2015)	- Trust in Medical Technology Scale – adapted from Trust in Physician Scale Anderson and Dedrick (1990) (11 items, strongly agree to strongly disagree scale).
Ishak and Nathan-Roberts (2015)	SEIPS 2.0 model:<br>- Elderly person's trust in the robot's ability with their life<br>- The elderly person's doctor trusting the robot<br>- The elderly person's family trusting the robot
Correia et al. (2016)	Not mentioned
Loghmani et al. (2019)	Custom open-ended questionnaire:<br>- Do you trust the robot?
Mo et al. (2017)	Trust in social robots:<br>- I believe advice from This when I don't know if it's correct.<br>- When uncertain, I believe This rather than myself.<br>- I have faith that This will provide the best solution.<br>- When This gives unusual advice, I'm confident it's correct.
Erebak and Turgut (2019)	- Trust in automation via checklist by Jian et al. (2000). 6-point scale (1 = strongly disagree, 6 = strongly agree). Questions not mentioned.
Pak et al. (2020)	- Trust in the robot in the scenario. Likert scale: 1 (not at all) to 7 (very much).
Fakhrhosseini et al. (2020)	- Trust in technology's ability to protect user privacy.
Harris and Rogers (2021)	- Trust in technology: "It's just like the one … the nurse puts on… probably FDA approved…"<br>- Trust in person: "Because I trust my doctor…"<br>- Conditional trust: "Once I know it's calibrated properly…"<br>- Lack of trust: "I don't trust it and I don't think I need it."
Fitter et al. (2020)	- I trust the robot.
Sorrentino et al. (2021)	- I would trust the robot's ability to perform the cognitive assessment.<br>- I think the robot would be too intrusive for my privacy.
Fracasso et al. (2022)	- The user's belief that a robot behaves with integrity and reliability.
Huang (2022)	- I feel the service provided by the hotel service robot is real.<br>- I think the service is clear and reliable.<br>- I feel it's trustworthy to use robots in hotels.<br>- I feel hotel robots have the necessary ability for customer service.
Lorusso et al. (2023)	- I would trust the Pharaon System if it gave me advice.<br>- I would follow the advice the Pharaon System gives me.

Continued on next page

Study	Questions
Zafrani et al. (2023)	Human-Robot Trust Scale (0% to 100% trust):<br>What percentage of the time will this robot...<br>- Act consistently<br>- Function successfully<br>- Malfunction<br>- Have errors<br>- Provide feedback<br>- Meet mission needs<br>- Provide appropriate information<br>- Communicate with people<br>- Perform exactly as instructed<br>- Follow directions<br>- Be dependable<br>- Be reliable<br>- Be unresponsive<br>- Be predictable
Rahman (2023)	- What is your level of trust in the virtual human based on her assistance in finding the missing object?<br>- List the factors (functions, attributes, etc.) that influence trust. Indicate their importance using a Likert scale from 1 (least important) to 5 (most important).
Aharony et al. (2024)	- I felt I could really trust this robot.<br>- I can trust the information provided by the robot.<br>- I would trust the robot if it were to give me advice.

REFERENCES

Aharony, N., Krakovski, M., and Edan, Y. (2024). A transparency-based action model implemented in a robotic physical trainer for improved hri. *J. Hum.-Robot Interact.* 14. doi:10.1145/3700598

Aly, H., Byrne, K. A., and Knijnenburg, B. (2024). Perceived trustworthiness of human vs. ai instructors in digital privacy education for older adults. In *Companion Proceedings of the 29th International Conference on Intelligent User Interfaces*. 107–112

Amin, M. S., Johnson, V. L., Prybutok, V., and Koh, C. E. (2024). An investigation into factors affecting the willingness to disclose personal health information when using ai-enabled caregiver robots. *Industrial Management & Data Systems* 124, 1677–1699

Anderson, L. A. and Dedrick, R. F. (1990). Development of the trust in physician scale: a measure to assess interpersonal trust in patient-physician relationships. *Psychological reports* 67, 1091–1100

Begum, M., Huq, R., Wang, R., and Mihailidis, A. (2015). Collaboration of an assistive robot and older adults with dementia. *Gerontechnology* 13, 405–419. doi:10.4017/gt.2015.13.4.005.00. Cited By :13 Export Date: 2 August 2022

Branyon, J. and Pak, R. (2015). Investigating older adults' trust, causal attributions, and perception of capabilities in robots as a function of robot appearance, task, and reliability. In *Proceedings of the Human Factors and Ergonomics Society Annual Meeting*. vol. 2015-January, 1550–1554. doi:10.1177/1541931215591335. Cited By :5 Export Date: 2 August 2022

Camilleri, A., Dogramadzi, S., and Caleb-Solly, P. (2022). Learning from carers to inform the design of safe physically assistive robots - insights from a focus group study. In *2022 17th ACM/IEEE International Conference on Human-Robot Interaction (HRI)* (IEEE Press), 703–707. doi:10.1109/HRI53351.2022.9889658

Cavallo, F., Limosani, R., Manzi, A., Bonaccorsi, M., Esposito, R., Di Rocco, M., et al. (2014). Development of a socially believable multi-robot solution from town to home. *Cognitive Computation* 6, 954–967. doi:10.1007/s12559-014-9290-z

Correia, F., Alves-Oliveira, P., Maia, N., Ribeiro, T., Petisca, S., Melo, F. S., et al. (2016). Just follow the suit! trust in human-robot interactions during card game playing. In *2016 25th IEEE international symposium on robot and human interactive communication (RO-MAN)* (IEEE), 507–512. doi:10.1109/ROMAN.2016.7745165

Daniele, K., Marcucci, M., Cattaneo, C., Borghese, N. A., and Zannini, L. (2019). How prefrail older people living alone perceive information and communications technology and what they would ask a robot for: Qualitative study. *Journal of Medical Internet Research* 21. doi:10.2196/13228. Cited By :6 Export Date: 2 August 2022

Do, H. M., Sheng, W., Harrington, E. E., and Bishop, A. J. (2021). Clinical screening interview using a social robot for geriatric care. *IEEE Transactions on Automation Science and Engineering* 18, 1229–1242. doi:10.1109/TASE.2020.2999203

Ejdys, J. (2022). Factors affecting trust in selected gerontechnologies: The case of poland. In *Proceedings of the Future Technologies Conference* (Springer), 592–601

Erebak, S. and Turgut, T. (2019). Caregivers' attitudes toward potential robot coworkers in elder care. *Cognition, Technology and Work* 21, 327–336. doi:10.1007/s10111-018-0512-0. Cited By :19 Export Date: 2 August 2022

Fakhrhosseini, S., Lee, C., Miller, J., Patskanick, T., and Coughlin, J. (2020). Older adults' opinion on social robot as companion. In *2020 29th IEEE International Conference on Robot and Human Interactive Communication (RO-MAN)*. 821–826. doi:10.1109/RO-MAN47096.2020.9223578. Cited By :1 Export Date: 2 August 2022

Fiorini, L., Pani, J., Rovini, E., Toccafondi, L., Calamida, N., Vignani, G., et al. (2023). Evaluating telepresence robot for supporting formal and informal caregivers in the care support service: A six-month case study. In *International Conference on Social Robotics* (Springer), 275–284

Fitter, N. T., Mohan, M., Kuchenbecker, K. J., and Johnson, M. J. (2020). Exercising with baxter: preliminary support for assistive social-physical human-robot interaction. *Journal of neuroengineering and rehabilitation* 17, 1–22

Fracasso, F., Buchweitz, L., Theil, A., Cesta, A., and Korn, O. (2022). Social robots acceptance and marketability in italy and germany: A cross-national study focusing on assisted living for older adults. *International Journal of Social Robotics* doi:10.1007/s12369-022-00884-z. Cited By :1 Export Date: 2 August 2022

Giorgi, I., Minutolo, A., Tirotto, F., Hagen, O., Esposito, M., Gianni, M., et al. (2023). I am robot, your health adviser for older adults: Do you trust my advice? *International Journal of Social Robotics* , 1–20

Gul, A., Turner, L. D., and Fuentes, C. (2024). An exploratory analysis of trust in socially assistive robot interactions with unpaid carers of older adults. In *2024 33rd IEEE International Conference on Robot and Human Interactive Communication (ROMAN)* (IEEE), 1630–1637

Harris, M. T. and Rogers, W. A. (2021). Developing a healthcare technology acceptance model (h-tam) for older adults with hypertension. *Ageing and Society* doi:10.1017/S0144686X21001069. Cited By :2 Export Date: 2 August 2022

Hoppe, J. A., Melkas, H., Pekkarinen, S., Tuisku, O., Hennala, L., Johansson-Pajala, R.-M., et al. (2022). Perception of society's trust in care robots by public opinion leaders. *International Journal of Human-Computer Interaction* doi:10.1080/10447318.2022.2081283. Export Date: 2 August 2022

Huang, T. (2022). What affects the acceptance and use of hotel service robots by elderly customers? *Sustainability* 14, 16102

Ishak, D. and Nathan-Roberts, D. (2015). Analysis of elderly human-robot team trust models. In *Proceedings of the Human Factors and Ergonomics Society Annual Meeting*. vol. 2015-January, 65–69. doi:10.1177/1541931215591014. Cited By :4 Export Date: 2 August 2022

Jian, J.-Y., Bisantz, A. M., and Drury, C. G. (2000). Foundations for an empirically determined scale of trust in automated systems. *International journal of cognitive ergonomics* 4, 53–71. doi:10.1207/S15327566IJCE0401_04

Kumar, S., Halloun, S., Itzhak, E., Tractinsky, N., Nimrod, G., and Edan, Y. (2022). Exploring the influence of culture and gender on older adults' perception of polite robots. In *2022 31st IEEE International Conference on Robot and Human Interactive Communication (RO-MAN)* (IEEE), 1038–1043

Lee, H., Kim, Y., and Bianchi, A. (2017). A survey on medical robotic telepresence design from the perspective of medical staff. *Archives of Design Research* 30, 61. doi:10.15187/adr.2017.02.30.1.61

Loghmani, M. R., Haider, C., Chebotarev, Y., Tsiourti, C., and Vincze, M. (2019). Effects of task-dependent robot errors on trust in human-robot interaction: A pilot study. In *2019 IEEE SmartWorld, Ubiquitous Intelligence & Computing, Advanced & Trusted Computing, Scalable Computing & Communications, Cloud & Big Data Computing, Internet of People and Smart City Innovation (SmartWorld/SCALCOM/UIC/ATC/CBDCom/IOP/SCI)*. 172–177. doi:10.1109/SmartWorld-UIC-ATC-SCALCOM-IOP-SCI.2019.00072

Lorusso, L., Mosmondor, M., Grguric, A., Toccafondi, L., D'Onofrio, G., Russo, S., et al. (2023). Design and evaluation of personalized services to foster active aging: The experience of technology pre-validation in italian pilots. *Sensors* 23, 797

Mann, J. A., MacDonald, B. A., Kuo, I.-H., Li, X., and Broadbent, E. (2015). People respond better to robots than computer tablets delivering healthcare instructions. *Computers in Human Behavior* 43, 112–117. doi:10.1016/j.chb.2014.10.029

Marin, A. L. and Lee, S. (2013). Interaction design for robotic avatars does avatar's aging cue affect the user's impressions of a robot? In *International Conference on Universal Access in Human-Computer Interaction* (Springer), 373–382

Mo, F., Zhou, J., and Yi, S. (2017). How to enhance intergenerational communication? the influence of family orientation and generation when using social robots as an intermediary. In *International Conference on Human Aspects of IT for the Aged Population* (Springer), 348–359

Newaz, F. and Saplacan, D. (2018). Exploring the role of feedback on trust for the robots used in homes of the elderly. In *Proceedings of the 10th Nordic conference on human-computer interaction*. 681–685. doi:10.1145/3240167.3240248. Cited By :3 Export Date: 2 August 2022

Ono, S., Obo, T., Kiong, L. C., and Kubota, N. (2015). Robot communication based on relational trust model. In *IECON 2015-41st Annual Conference of the IEEE Industrial Electronics Society* (IEEE), 005335–005338. doi:10.1109/IECON.2015.7392941

Pak, R., Crumley-Branyon, J. J., de Visser, E. J., and Rovira, E. (2020). Factors that affect younger and older adults' causal attributions of robot behaviour. *Ergonomics* 63, 421–439. doi:10.1080/00140139.2020.1734242. Cited By :3 Export Date: 2 August 2022

Pascher, M., Kronhardt, K., Franzen, T., Gruenefeld, U., Schneegass, S., and Gerken, J. (2022). My caregiver the cobot: Comparing visualization techniques to effectively communicate cobot perception to people with physical impairments. *Sensors* 22. doi:10.3390/s22030755. Cited By :1 Export Date: 2 August 2022

Piasek, J. and Wieczorowska-Tobis, K. (2018). Acceptance and long-term use of a social robot by elderly users in a domestic environment. In *2018 11th international conference on human system interaction (HSI)*. 478–482. doi:10.1109/HSI.2018.8431348

Poulsen, A., Burmeister, O. K., and Kreps, D. (2018). The ethics of inherent trust in care robots for the elderly. In *IFIP International Conference on Human Choice and Computers* (Springer), 314–328

Rahman, S. M. (2023). Human trust between real and virtual agents for a real-world assistive task. In *International conference on WorldS4* (Springer), 491–499

Rossi, S., Santangelo, G., Staffa, M., Varrasi, S., Conti, D., and Nuovo, A. D. (2018). Psychometric evaluation supported by a social robot: Personality factors and technology acceptance. In *2018 27th IEEE international symposium on robot and human interactive communication (RO-MAN)*. 802–807. doi:10.1109/ROMAN.2018.8525838

Sorrentino, A., Mancioppi, G., Coviello, L., Cavallo, F., and Fiorini, L. (2021). Feasibility study on the role of personality, emotion, and engagement in socially assistive robotics: A cognitive assessment scenario. *Informatics* 8. doi:10.3390/informatics8020023. Cited By :1 Export Date: 2 August 2022

Stuck, R. E. and Rogers, W. A. (2017). Understanding older adult's perceptions of factors that support trust in human and robot care providers. In *Proceedings of the 10th International Conference on PErvasive Technologies Related to Assistive Environments* (Association for Computing Machinery), 372–377. doi:10.1145/3056540.3076186

Tan, S.-H., Yap, Y.-Y., Tan, S.-K., and Wong, C.-K. (2024). Informal caregivers' perception of assistive robots in eldercare. *Journal of Open Innovation: Technology, Market, and Complexity* 10, 100234

Ting, K. L. H., Voilmy, D., Iglesias, A., Pulido, J. C., Garcia, J., Romero-Garces, A., et al. (2017). Integrating the users in the design of a robot for making comprehensive geriatric assessments (cga) to elderly people in care centers. In *2017 26th IEEE International Symposium on Robot and Human*

Interactive Communication (RO-MAN). vol. 2017-January, 483–488. doi:10.1109/ROMAN.2017.8172346. Cited By :7 Export Date: 2 August 2022

Torta, E., Werner, F., Johnson, D. O., Juola, J. F., Cuijpers, R. H., Bazzani, M., et al. (2014). Evaluation of a small socially-assistive humanoid robot in intelligent homes for the care of the elderly. *Journal of Intelligent & Robotic Systems* 76, 57–71. doi:10.1007/s10846-013-0019-0

Wald, S., Puthuveetil, K., and Erickson, Z. (2024). Do mistakes matter? comparing trust responses of different age groups to errors made by physically assistive robots. In *2024 33rd IEEE International Conference on Robot and Human Interactive Communication (ROMAN)* (IEEE), 373–380

Wonseok, J., Woo, K. Y., and Yeonheung, K. (2021). Who made the decisions: Human or robot umpires? the effects of anthropomorphism on perceptions toward robot umpires. *Telematics and Informatics* 64, 101695

Yan, R., Tee, K. P., Chua, Y., and Huang, Z. (2013). A user study for an attention-directed robot for telepresence. In *International Conference on Smart Homes and Health Telematics* (Springer), 110–117

Zafrani, O., Nimrod, G., and Edan, Y. (2023). Between fear and trust: Older adults' evaluation of socially assistive robots. *International Journal of Human-Computer Studies* 171, 102981

Zhang, X., Zhang, P., Zeng, X., Wang, Y., and Chi, C.-H. (2022). sauth: a hierarchical implicit authentication mechanism for service robots. *Journal of Supercomputing* doi:10.1007/s11227-022-04472-w. Export Date: 2 August 2022
